# Bone-Marrow-Derived Mesenchymal Stem Cells, Their Conditioned Media, and Olive Leaf Extract Protect against Cisplatin-Induced Toxicity by Alleviating Oxidative Stress, Inflammation, and Apoptosis in Rats

**DOI:** 10.3390/toxics10090526

**Published:** 2022-09-06

**Authors:** Mahrous A. Ibrahim, Athar M. Khalifa, Alaa A. Mohamed, Rania A. Galhom, Horeya E. Korayem, Noha M. Abd El-Fadeal, Ahmed Abd-Eltawab Tammam, Mohamed Mansour Khalifa, Osama S. Elserafy, Rehab I. Abdel-Karim

**Affiliations:** 1 Forensic Medicine and Clinical Toxicology, College of Medicine, Jouf University, Sakaka 41412, Saudi Arabia; 2 Forensic Medicine and Clinical Toxicology Department, Faculty of Medicine, Suez Canal University (SCU), Ismailia 41522, Egypt or; 3 Pathology Department, College of Medicine, Jouf University, Sakaka 41412, Saudi Arabia; 4 Medical Biochemistry Division, Pathology Department, College of Medicine, Jouf University, Sakaka 41412, Saudi Arabia; 5 Medical Biochemistry Department, Faculty of Medicine, Beni-Suef University, Beni-Suef 62514, Egypt; 6 Human Anatomy and Embryology Department, Faculty of Medicine, Suez Canal University (SCU), Ismailia 41522, Egypt; 7 Center of Excellence in Molecular and Cellular Medicine (CEMCM), Faculty of Medicine, Suez Canal University (SCU), Ismailia 41522, Egypt; 8 Human Anatomy and Embryology Department, Faculty of Medicine, Badr University in Cairo (BUC), Cairo 11829, Egypt; 9 Histology and Cell Biology Department, Faculty of Medicine, Suez Canal University (SCU), Ismailia 41522, Egypt; 10 Medical Biochemistry and Molecular Biology Department, Faculty of Medicine, Suez Canal University (SCU), Ismailia 41522, Egypt; 11 Oncology Diagnostic Unit, Faculty of Medicine, Suez Canal University (SCU), Ismailia 41522, Egypt; 12 Physiology Department, College of Medicine, Jouf University, Sakaka 41412, Saudi Arabia; 13 Physiology Department, Faculty of Medicine, Beni-Suef University, Beni-Suef 62514, Egypt; 14 Human Physiology Department, Faculty of Medicine, Cairo University, Cairo 11562, Egypt; 15 Human Physiology Department, College of Medicine, King Saud University, Riyadh 11472, Saudi Arabia; 16 Forensic Medicine and Clinical Toxicology Department, Faculty of Medicine, Cairo University, Cairo 11562, Egypt; 17 Criminal Justice and Forensic Sciences Department, King Fahd Security College, Riyadh 11451, Saudi Arabia

**Keywords:** apoptosis, BM-MSCs, cisplatin toxicity, genotoxicity, oxidative stress, PCR, immunotoxicity, nephrotoxicity, hepatotoxicity

## Abstract

Background: Hepatic and renal damage is a cisplatin (Cis)-induced deleterious effect that is a major limiting factor in clinical chemotherapy. Objectives: The current study was designed to investigate the influence of pretreatment with olive leaf extract (OLE), bone-marrow-derived mesenchymal stem cells (BM-MSC), and their conditioned media (CM-MSC) against genotoxicity, nephrotoxicity, hepatotoxicity, and immunotoxicity induced by cisplatin in rats. Methods: The rats were randomly divided into six groups (six rats each) as follows: Control; OLE group, treated with OLE; Cis group, treated with a single intraperitoneal dose of Cis (7 mg/kg bw); Cis + OLE group, treated with OLE and cisplatin; Cis + CM-MSC group, treated with BM-MSC conditioned media and Cis; and Cis + MSC group, treated with BM-MSC in addition to Cis. Results: Cis resulted in a significant deterioration in hepatic and renal functions and histological structures. Furthermore, it increased inflammatory markers (TNF-α, IL-6, and IL-1β) and malondialdehyde (MDA) levels and decreased glutathione (GSH) content, total antioxidant capacity (TAC), catalase (CAT), and superoxide dismutase (SOD) activity in hepatic and renal tissues. Furthermore, apoptosis was evident in rat tissues. A significant increase in serum 8-hydroxy-2-deoxyguanosine (8-OH-dG), nitric oxide (NO) and lactate dehydrogenase (LDH), and a decrease in lysozyme activity were detected in Cis-treated rats. OLE, CM-MSC, and BM-MSC have significantly ameliorated Cis-induced deterioration in hepatic and renal structure and function and improved oxidative stress and inflammatory markers, with preference to BM-MSC. Moreover, apoptosis was significantly inhibited, evident from the decreased expression of Bax and caspase-3 genes and upregulation of Bcl-2 proteins in protective groups as compared to Cis group. Conclusions: These findings indicate that BM-MSC, CM-MSC, and OLE have beneficial effects in ameliorating cisplatin-induced oxidative stress, inflammation, and apoptosis in the hepatotoxicity, nephrotoxicity, immunotoxicity, and genotoxicity in a rat model.

## 1. Introduction

Anticancer drugs have been increasingly used over the last decades due to the remarkable increase in cancer prevalence worldwide. Chemotherapeutic agents have various and serious side effects due to their non-selective action on body cells. Although they aim to damage the abnormally proliferating neoplastic cells, they may damage normal body cells as well, especially the rapidly dividing cells, leading to serious health hazards [1]. Cisplatin is an extensively used, effective chemotherapeutic agent. It is used against a wide range of malignant tumors targeting testicles, ovaries, lungs, and other organs [2]. 

The mechanism of action of cisplatin includes DNA damage through adducts formation, leading eventually to apoptosis and cancerous cell death [3,4]. Several studies have reported cisplatin-induced side effects that were responsible for limiting its administration therapeutically [5,6,7,8]. Several researchers have investigated the role of Cis in elevating oxidative stress, and producing genotoxicity, cytotoxicity, and reproductive impairment [1,9,10]. Consequently, the protective role of antioxidants in combating side effects of cisplatin has been investigated [1,9,10]. Several studies confirmed Cis-induced hepatotoxicity [11,12,13,14] and nephrotoxicity [12,15,16,17]. The mechanisms underlying liver injury due to Cis intake are still not fully understood. However, liver injury may be attributed to the Cis-induced oxidative stress and apoptosis [18]. Cisplatin has been proven to exert renal damage in the form of tubular cells necrosis [19]. Twenty percent of Cis-treated patients suffered from acute renal failure [20]. Cis is eliminated mainly through glomerular filtration and tubular secretion, and it accumulates in proximal and distal tubules cells [21]. It exerts renal damage through complex pathways that include oxidative stress, mitochondrial dysfunction, endoplasmic reticulum dysfunction, DNA damage, and apoptosis [17,22]. Inflammation has been reported to play a role in Cis-induced hepatic [11,14] and renal damage [15,16,17]. Various studies have addressed the potential protective effects of some natural and synthetic substances against Cis-induced renal and hepatic toxicity [23,24,25]. 

Mesenchymal stem cells (MSCs) therapy is a new therapy with high and promising potentials. The MSCs have shown promising results in treatment of neurological, autoimmune, hepatic, cardiac, and kidney diseases [26,27]. The success rate of their use depends to some extent on the donor, the origin of their cells, and their cultivation environment [28,29]. The researchers attributed the protective role of those cells to their possible antioxidative, anti-inflammatory, and anti-apoptotic action [29], besides their immunomodulatory effect [30]. Bone-marrow-derived MSCs (BM-MSCs) are among the most-used cell types in clinical trials and experimental research. They are currently being studied and tested for the treatment of a wide range of diseases [31]. MSCs exhibited a protective role against damage in some organs, including the myocardium, cartilage, and nervous tissue [29]. BM-MSCs were able to ameliorate Cis-induced testicular damage through suppression of oxidative stress, down-regulation of inflammatory mediators (iNOS and TNF-α), and downregulation of pro-apoptotic markers (caspase-3 and Bax), in addition to their action in differentiation and replacing damaged tissue [32].

Some studies have investigated the regenerative ability of MSCs for treating acute kidney impairment [33], and other studies addressed their regenerative power in liver disease [34]. Based on the principle of the paracrine signaling mechanism, mesenchymal-stem-cell conditioned media (CM-MSC), also referred to as the secretome of the MSCs, are considered a rich source of the paracrine factors, and are being studied extensively for a wide range of regenerative therapeutic strategies such as myocardial infarction, stroke, bone regeneration, hair growth, and wound healing. Many reviews have appraised the role of paracrine factors, which are secreted by stem cells, as the driving force behind their regenerative capacity [35]. Studies have reported that MSCs, through their paracrine action, downregulated fibrogenic growth factors [36], iNOS, and pro-inflammatory cytokines. In addition, they upregulated the anti-inflammatory and anti-apoptotic factors [37]. Human umbilical CM-MSC was found to contain exosomes that could be isolated and used to resist Cis-induced oxidative stress on renal tissue [38].

Olive leaves, an agricultural waste product, are obtained during the process of harvesting olive fruits [39]. Olive leaf extract (OLE) is a natural product commonly used on a large scale [40]. It contains phenolic compounds, flavonoids, and tocopherol [41]. Olive leaf extract (OLE) has proven multiple health benefits including anti-microbial anti-inflammatory, antihypertensive, and antioxidant properties [39,42]. It has exerted gastroprotective effect against Hcl/ethanol-induced gastritis either directly through decreasing the expression of NF-kB, TNF-α, IL-β, and cyclo-oxygenase-2 (COX-2) in gastric tissue, or indirectly through its antioxidant activity [43]. Oleuropein is the main compound present in OLE, and it has proven anti-inflammatory and antioxidant functions [39]. It has shown promising protective results against cisplatin-induced renal, cytotoxic, and DNA damage [44]. Phenolic compounds in olive fruit extract and oleuropein ameliorated deltamethrin-induced hepatorenal toxicity [45]. Extra virgin olive oil (EVOO) contains oleic acid which prevents apoptosis in liver cells, and it possess antioxidant abilities that provided hepato-protective functions [46]. Studies have acknowledged the synergistic action between phenolic and flavonoids constituents existing in OLE [47].

To our knowledge, few studies have reported on the anti-inflammatory and anti-apoptotic effect of OLE against Cis-induced renal damage [44]. Furthermore, no studies have addressed the protective role of BM-MSCs, their conditioned media, or OLE against Cis-induced hepatotoxicity. Due to numerous technological innovations in medical science, the medical industry can present various alternative therapies to treat organ toxicity and degenerative diseases. However, some people are willing to undergo traditional therapies, including natural products like OLE. The current study was tailored to investigate and compare the protective effect of OLE, CM-MSC, and BM-MSC against cisplatin induced genotoxicity, cytotoxicity, immunotoxicity, nephrotoxicity, and hepatotoxicity by investigating specific biochemical and genetic markers, and with histopathological, histochemical, and immunohistochemical examination of liver, kidneys, spleen, and thymus gland.

## 2. Materials and Methods

### 2.1. Chemicals 

Cisplatin (Cis-10 mg per vial) used in the current study was manufactured by Mylan Institutional LLC, Rockford, IL, USA.

Olive leaf extract (OLE) was prepared before carrying out the experiment. To prepare the extract, fresh (*Olea europaea* L.) olive leaves were harvested from a local area in Aljouf province, (Saudi Arabia). The collected leaves were air-dried after washing, and then by utilizing an electric grinder, the dried leaves were ground to powder. This powder was soaked in 80% ethanol for 24 h to prepare the extract. At a temperature of less than 45 °C and by using rotary evaporator, the resulting mixture was filtered and dried. The dried residue was utilized in rat treatments. 

BM-MSCs were harvested, cultured, and expanded to Passage 2 (p2) in the tissue culture lab of the Center of Excellence in Molecular and Cellular Medicine (CEMCM), Faculty of Medicine, Suez Canal University.

The other chemicals and kits used in the experiment were acquired from “Bio diagnostic Company, Giza, Egypt” and “Sigma Chemical Company, St. Louis, MO, USA”.

### 2.2. Experimental Animal 

Thirty-six male Wistar rats, 200–230 g in body weight and 12 to 14 weeks old, were used in the study. The animals were housed in large individual cages in an air-conditioned room. Healthy animals were left to acclimatize for 14 days. They were housed under standard conditions, with an optimum temperature of 24 ± 2 °C and 45 ± 5% humidity. Rats were exposed to 12 h dark and 12 h light cycles, fed standard chow diet, and provided water ad libitum. Our experiment was performed in compliance with the United Stated National Institute of Health publications concerning the Guide for Care and Use of Laboratory Animals (No. 85–23, revised 1996). The study was approved by the Local Committee of Bioethics (LCBE), Jouf University, Aljouf, Saudi Arabia (#02-08-42).

### 2.3. Experimental Design

**Scheme 1:**
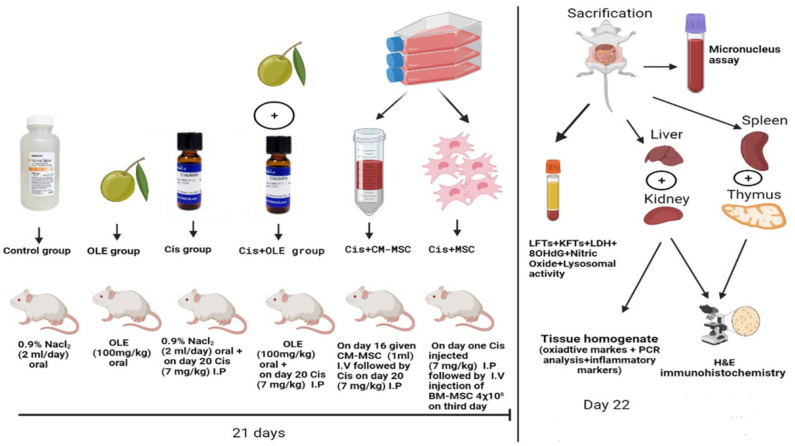
A schematic diagram showing the experimental design.

Rats were randomly allocated into six equal groups (*n*= 6/group) as follows (Scheme 1): 

Group 1 (Control): By oral gastric gavage, the rats were administered normal saline (NS, 0.9% NaCl) in similar volume (2 mL/day) for 21 days, and they were served as a normal control group.

Group 2 (OLE): Rats received OLE (100 mg/kg of body weight) once daily for 21 days by oral administration [48,49].

Group 3 (Cis): Rats were treated by oral gastric gavage with normal saline (NS, 0.9% NaCl) for 21 consecutive days. On day 20, these rats were given cisplatin (7 mg/kg of body weight) freshly dissolved in saline (0.9% NaCl) by single intraperitoneal (IP) injection [1,17].

Group 4 (Cis + OLE): Rats received oral OLE (100 mg/kg of body weight) once daily for 21 days. Then, these rats were given cisplatin (7 mg/kg of body weight), freshly dissolved in saline (0.9% NaCl) on day 20, by single intraperitoneal (IP) injection.

Group 5 (Cis + CM-MSC): The rats were subjected to induction of toxicity using the same protocol of group 3, and they received 1 mL of BM-MSCs conditioned media by intravenous injection (IV) for four consecutive days prior to toxicity induction [50].

Group 6 (Cis + MSC): The rats were subjected to induction of toxicity using the same protocol of group 3, but the induction took place on day one of the study, and then the animals received IV injection of BM-MSCs at a dose of 4X10^6^ cells on the 3rd day of induction and were sacrificed on the 21st day [51].

Cisplatin can be administered in humans as an injectable agent intravenously or intra-arterial. Dosing runs from 20 mg/m^2^ to 100 mg/ m^2^ in 21- to 28-day cycles, depending on the type of malignancy [52]. However, the used dose of cisplatin in our work was chosen according to its induction of hepatic and renal toxicity as implicated by Yousef et al. and Salem et al. [1,17].

### 2.4. Stem Cells Isolation, Culture, and Expansion

Rat BM-MSCs were isolated from the tibias and femurs of donner male rats under sterile aseptic condition. Briefly, after euthanasia, long bones were dissected free of soft tissues, shaved by surgical blades, and cut open at both ends with scissors. Then, bone marrow was harvested by flushing out from the bone cavity with DMEM (Lonza, Basel, Switzerland) using a 22-gauge syringe. Bone marrow was vigorously pipetted repeatedly to produce a single-cell suspension, followed by filtration through a 70 μm nylon mesh filter. After being centrifuged at 1800 rpm for 10 min, the supernatant of the single-cell suspension was discarded. Cell pellets were suspended with complete media and cultured in T25 flasks (CELLSTAR-Greiner bio-one) containing complete media; composed of 89% DMEM, 10% fetal bovine serum (FBS), and 1% penicillin/streptomycin (PS) (1:1) (Lonza, Verviers, Belgium). The medium was refreshed every 2–3 days. All cultures were incubated at 37 °C in a 5% CO_2_ under humidified environment. The non-adherent cells were removed by changing the culture medium. When the adherent cells grew to 85–95% confluence, the culture was trypsinized by 0.25% trypsin/EDTA (Gibco, Grand Island, NY, USA) and expanded through three passages (P1–P2). Cells of P2 were used for characterization and transplantation [53].

### 2.5. Phenotypic Characterization of BM-MSCs

Immunophenotypic assessment of the BM-MSCs surface markers was done in the Oncology Diagnostic Unit, Faculty of Medicine, Suez Canal University. After trypsinization, in the 2nd passage (p2), cells were counted using trypan blue exclusion test, and 5 × 10^5^ of MSCs were labelled with fluorochrome-conjugated mouse anti-rat antibodies CD90 FITC (fluorescein isothiocyanate), CD45 PerCP (peridinin–chlorophyll–protein), CD34 PE (phycoerythrin), and CD73 APC (alkaline phosphatase conjugate) for 20 min at 4 °C in the dark. All CDs were purchased from BD Biosciences, USA. Also, we used unstained MSCs as controls. The tubes were centrifuged for 5 min at 150× *g* at room temperature. Cell pellets were washed with 1× phosphate buffer saline (PBS) in a dark room and centrifuged. After that, cell pellets were resuspended using 2 mLof PBS and analyzed for surface marker profile using an Partec-CyFlow^®^ ML instrument. Data acquisition, analysis, and display were performed with FloMax^®^software (Partec GmbH, Münster, Germany).

### 2.6. Assessment of Viability, Cell Count and Transplantation of BM-MSCs 

Trypan blue exclusion assay was used to assess the viability and the count of cells at the beginning and at the end of each passage (P0–P2), and prior to transplantation. Trypan Blue stain 0.4% (Lonza, Basel, Switzerland) and hemocytometer chambers (MARIENFIELD, Thuringia, Germany) were used for this purpose. Cells of P2 were washed after trypsinization with phosphate-buffered saline (PBS), (Lonza, Basel, Switzerland), and suspended in DMEM, and adjusted to be 4 × 10^6^ cells/mL Each rat of group 6 received 1 mL of this suspension by IV injection in the tail vein. 

### 2.7. Collection and Preparation of BM-MSCs Conditioned Media (CM-MSC)

The same method of Angoulvant et al. [54] was used to collect and prepare BM-MSC conditioned media. It was obtained by incubating freshly trypsinized BM-MSCs of P2 in serum-deprived fresh DMEM (Gibco) for 8 h in an incubator with 5% CO_2_. The supernatant was collected from the tissue culture vessels and centrifuged at 1800 rpm for 5 min to remove any detached cells. Each rat of group five received 1 mL of freshly prepared CM-MSC through IV rout daily for four consecutive days prior to toxicity induction. 

### 2.8. Tissues Harvesting and Samples Collection

At the end of the experiment (day 21), study animals fasted overnight. On the next day, they were inspected, weighted up, and sacrificed. Scarification was done ethically under anesthesia, using cervical dislocation. Following that, blood was gathered, then centrifuged, to separate serum, for 10 min (3000 r.p.m). Sera were preserved under −80 °C and kept for measurement of the hepatic and renal parameters. The liver, kidney, spleen, and thymus were cautiously extracted from each rat body cavity, then cleared and cleaned from adherent tissues. Liver and kidney weights were measured for each rat. A piece from the liver and kidney was homogenized and used for oxidative and pro-inflammatory markers assessment and PCR analysis. After washing the collected tissue pieces, they were thoroughly rinsed using ice-cold saline. Afterwards, they were dried and kept at −20 °C for subsequent analysis. To perform the histopathological examination and immunohistochemical investigations, formalin solution (10%) was used for fixation of the remaining pieces of the four collected organs.

### 2.9. Assessment of the Body, Liver, and Kidney Weights 

Rat total body weight was measured at the beginning and end of the experiment, and liver and kidney weights were measured immediately after scarification, at the end of the experiment, using a sensitive balance to evaluate the change in liver and kidney weights in relation to the entire rat body weight. The liver and kidney mean weights were estimated as mg/g body weight of rats.

Relative body weight (RBW) = (final body weight/initial body weight) × 100

Relative liver weight (RLW) = (liver weight/final body weight) × 100

Relative kidney weight (RKW) = (kidney weight/final body weight) × 100

### 2.10. Assessment of Liver Necrosis and Hepatic Functions Markers

Liver enzymes, alanine aminotransferase (ALT), and aspartate aminotransferase (AST) were measured in serum as U/L according to Reitman and Frankel’s method [55]. Otto et al. method [56] was used to measure alkaline phosphatase (ALP) in U/L serum. Both albumin and total proteins were measured in serum as g% using Northam and Kingsley and Pinnell methods [57,58], respectively. Serum level of bilirubin was determined as mg/dL according to Perry et al. [59] method.

### 2.11. Measurement of Renal Function Markers

For biochemical analysis, levels of urea and creatinine were assessed according to the methods described by Banday et al. [60] and Buchanan [61], respectively. 

### 2.12. Estimation of the Markers of Oxidative Stress and Antioxidants in Hepatic and Renal Tissues 

Glutathione (GSH) concentration, the activity of superoxide dismutase enzyme (SOD), catalase (CAT) activity, and malondialdehyde (MDA) levels were estimated in both hepatic and renal tissues homogenates according to Beutler et al. technique [62], Masayasu and Hiroshi technique [63], Aebi method [64], and Kei technique [65], respectively. Total antioxidant capacity (TAC) was assessed using a commercial ELISA kit (MyBioSource Co., San Diego, CA, USA). All steps were carried out according to the manufacturer’s protocol.

### 2.13. Assessment of the Levels of Pro-Inflammatory Markers (IL-1β, IL-6, and TNF-α)

The Rat ELISA commercial kits (MyBioSource Co., San Diego, CA, USA) were utilized to assess interleukins 6 and 1β (IL-6 and IL-1β) and tumor necrosis factor-α (TNF-α) in hepatic and renal tissue homogenate.

### 2.14. Quantification of 8-Hydroxy-2-Deoxyguanosine (8-OH-dG) Level in Serum

The OxiSelect^TM^ “Oxidative DNA Damage ELISA Kit”(Cell Biolabs, Inc., San Diego, CA, USA), with 100 pg/mL-20 ng/mL detection sensitivity range, was used to quantify 8-OH-dG in serum using Breton et al. method [66]. The quantity of 8-OH-dG in unknown samples was determined by comparing its absorbance with that of a known 8-OH-dG standard curve. 

### 2.15. Assessment of Lactate Dehydrogenase Enzyme (LDH) Activity

A ready-made reagent kit (Bio-Med Diagnostic, White City, OR, USA) was used to assess LDH activity and evaluate Cis-induced cell damage. The assessment was performed according to Kachmar and Moss [67] method, and its level was expressed as µ/L.

### 2.16. Assessment of Lysozyme Activity 

The turbidometric method of Ellis [68] described the measurement of lysozyme activity in serum. Twenty-five µL of each serum sample were piled up to the plate wells that contained a 1% phosphate buffer saline diluted agarose gel in which Micrococcus lysodeikticus cell were scattered. After 24 h, measurement of the clear zone diameter that was created around the wells was carried out. The standard lysozyme through logarithmic curve was used to obtain the concentration levels of lysozyme.

### 2.17. Assessment of Nitric Oxide (NO)

The method described by Rajaraman et al. [69] was applied to measure NO serum levels, utilizing Griess reagent. Equal volumes of serum and Griess reagent (50 µL each) were incubated in a 96 flat-bottomed microtiter plate well (at 27 °C for 10 min). Afterwards, the researchers recorded optical density with an ELIZA reader (at wavelength 570 nm). A standard curve for Na nitrite was used to calculate nitrite concentration. 

### 2.18. Micronucleus Assay

Drops of whole blood obtained by capillary tube from orbital vessels, just prior to scarification, were obtained and smeared directly on slides. Afterwards, we left the slides to dry in the air for 24 h, used acetone/methanol solution (1:1 for 10 min) to fix them, then they were stained with 10% Giemsa stain. Finally, we examined the slides using an oil immersion lens (1000×) to detect micronuclei in erythrocytes. The number of micro-nucleated cells was evaluated/1000 cells/each group of rats [70].

### 2.19. Histopathological Investigation

To detect the changes in histopathology of the tested organs, 10% formalin was used to fix all the harvested tissues samples for 24 h. Then, ethyl alcohol with ascending grades (70, 80, 95, and 100%) was used for standard dehydration of the samples. Afterwards, xylene was used for clearing the tissue samples before embedding them in paraffin-wax. Then, a microtome was used to cut the sections (5 µm) before its staining with hematoxylin and eosin (H&E). Furthermore, the liver and kidney sections were stained with Masson’s trichrome and PAS stains, respectively. Then, the tissue slides were inspected, examined, and photographed [71].

### 2.20. Immunohistochemical Investigation

Immuno-expression of cytokeratin in the thymus, PCNA in the spleen, and P53 in liver and kidney tissues were assessed according to Ramos et al. [72]. Before processing, deparaffinization of the paraffin sections was done. Then, antigen retrieval and incubation with the primary antibody at 4 °C were carried out against PCNA, P53, and cytokeratin antibodies (Abcam, Cambridge, UK). Afterwards, incubation of the sections was done with peroxidase-conjugated secondary antibody. To visualize the peroxidase, diaminobenzidine (DAB) was utilized. Sections were then counterstained with Mayer’s hematoxylin, washed in running water, dehydrated in graded ethanol, and mounted.

### 2.21. Morphometric Analysis 

For quantification of data, the area percentage of P53 antibody expression in liver and kidney sections besides the optical density of PCNA and cytokeratin antibodies expression in spleen and thymus respectively were assessed by analyzing image using “Image J 1.49 v/Java 1.6.0_244 (64-bit)” (NIH, Bethesda, MA, USA). Slides were stained and magnified (×400) to be used. Afterwards, we examined at least five non-overlapping sections/animals. The area percentage of the optical density of antibodies expression was measured per each section. The average percentage of stained cells was assessed per animal and averaged for each group. Prior to each analysis, a calibration system was applied to convert image pixels into micrometer units. The mean area percentage of collagen fibers in the liver sections stained with Masson Trichrome was assessed using the same method.

### 2.22. Tracking of Donner Male BM-MSCs in Female Tissues

Genomic DNAs were extracted from each group’s liver, kidney, spleen, and thymus gland using DNA mini^®^Kit (Qiagen, Hilden, Germany). The sex determination region on the Y chromosome (sry) gene was checked in the tissues of recipient female rats using a polymerase chain reaction (PCR) assay. Primer sequences for the sry gene are (FORWARD 5′-CATCGAAGGGTTAAAGCCA-3’, REVERSE 5’-ATAGTGTAGGTTGTTGTTGTCC-3′) and it was used to amplify a 104 bp employing the following PCR conditions: 94 °C for 4 min, 35 cycles of 94 °C for 50 s, 60 °C for 30 s, and 72 °C for 1 min, and a 7 min final extension at 72 °C. PCR products were electrophoresed on a 2% agarose gel, stained with ethidium bromide, and visualized by autoradiography [73,74].

### 2.23. PCR Analysis for Bcl-2, Bax, and Caspase-3 Genes

RNA was purified from homogenized liver and kidney tissues (0.5 g tissue: *n* = 6/group) following the manufacturer’s instructions of miRNeasy Mini Kit (Cat. no. 217004, Qiagen, Hilden, Germany). RNA concentrations and purity were evaluated at OD260/280 nm using NanoDrop One/Once Microvolume UV-Vis Spectrophotometers (Thermo Fisher Scientific, Waltham, MA, USA). Fifty micrograms of total RNA were reverse transcribed into complementary DNA (cDNA) using QuantiTect Reverse Transcription Kit (Cat. no. 205311, Qiagen, Hilden, Germany). Synthesized cDNA was stored at −20 °C until use in PCR analysis. Table 1 showed the specific primers which were used to evaluate the expression of Bax, Bcl-2, and caspase-3 genes. Real-time PCR analysis was completed using StepOne™ Real-Time PCR System (Cat. no. 4376357, Thermo Fisher Scientific, Swindon, UK), and the reaction mix was prepared using 10 μLof *HERA* SYBR^®^ Green qPCR master mix (Willowfort, Birmingham, UK), 15 pmol of each primer, and 50 ng of cDNA. PCR cycles were initial denaturation at 95 °C for 5 min, followed by 35 cycles of denaturation at 95 °C for 30 s, annealing at 58 °C for 30 s, and extension at 72 °C for 30 s. The relative RNA expression for genes was determined using ΔΔCt method, and the expression was normalized to the expression of the β-actin gene [75].

### 2.24. Statistical Analysis

Statistical Package for Social Sciences (SPSS) software, version 22 for windows (SPSS, Inc., Chicago, IL, USA), was applied for statistical analysis. Mean ± standard deviation (SD) was calculated and expressed for the numerical data found in the experiment. The figures and tables legends include the number of rats/each group. The homogeneity of variance among the treated groups was confirmed using Bartlett’s test. Afterwards, one-way analysis of variance (ANOVA) followed by Student–Newman–Keuls test were performed to assess the mean differences between the study groups/each parameter separately. Values of *p* < 0.05 were considered statistically significant.

## 3. Results

The rats were observed throughout the experiments and no mortalities were noticed in the tested and control groups.

### 3.1. The Effect of Cis, OLE, CM-MSC, and MSC on Body, Liver, and Kidney Weights

The used rats were matched in their body weight at the start of the study. The final body weights, relative body weight (RBW), liver weight, and relative liver weight (RLW) exhibited significant (*p* < 0.05) reduction in Cis-treated group than the control. However, there is a significant (*p* < 0.05) increase in kidneys and relative kidney weight (RKW) in Cis-treated group than the control group. Administration of OLE, CM-MSC, or MSC with Cis resulted in significant (*p* < 0.05) increase in final body weight than Cis-treated group. The administration of the three protective agents showed significant amelioration of the liver and kidneys weights towards the control level with preference to Cis + MSC group (Table 2).

### 3.2. The Effect of Cis, OLE, CM-MSC, and MSC on Liver and Renal Functions Markers

The statistics showed that single IP injection of cisplatin significantly worsened both liver and kidney functions when compared to the control group. This effect was evidenced by significant increases in serum ALT, and ALP and total bilirubin were associated with a decrease in serum total protein (TP) and albumin. Significant elevations in serum levels of urea and creatinine were also detected (Table 3). Cisplatin-induced rises in serum creatinine and urea levels were markedly reversed by treatment with OLE, CM-MSC, or MSC. In addition, these different treatment modalities impacted reversing cisplatin-induced changes in ALP, AST, and ALT levels in the serum with TP, albumin, and bilirubin. Also, the data indicate that MSC significantly (*p* < 0.05) reversed the deteriorated liver and kidney function slightly better than CM-MSC and OLE when given with cisplatin.

### 3.3. The Effect of Cis, OLE, CM-MSC, and MSC on Liver and Renal Oxidative/Antioxidants Status

The liver and kidney tissues of Cis-treated rats exhibited a substantial (*p* < 0.05) decrease in CAT, SOD, GSH, and TAC levels and an increase in MDA compared to the control group results. CAT, SOD, GSH, and TAC levels in the liver and kidneys were significantly higher in the groups that received OLE, CM-MSC, and MSC in adjunct to Cis, while MDA was significantly lower. Furthermore, the data showed that in the liver tissues, the activity of CAT, SOD, GSH, and TAC in the Cis + MSC treated group significantly increased in their levels, approaching those of the control group. The same occurs in the kidney tissues; the activity of CAT, SOD, GSH, and TAC showed a significant increase in their levels, approaching those of the control group. Cis + MSC depicted an increase in kidney TAC more than Cis + CM-MSC and Cis + OLE. Meanwhile, Cis + MSC significantly reduced MDA levels in liver and renal tissues compared to Cis + CM-MSC and Cis + OLE (Table 4).

### 3.4. The Effect of Cis, OLE, CM-MSC, and MSC on Liver and Renal Inflammatory Markers

In the liver and kidney tissues, we found that Cis injection significantly increased the levels of inflammation-related markers (IL-1β, IL-6, and TNF-α), suggesting that it triggers the release of inflammatory cytokines within these tissues. However, when Cis combined with OLE, CM-MSC, or MSC, the inflammatory markers showed a significant decrease, especially in the Cis + MSC group (Table 5).

### 3.5. The Effect of Cis, OLE, CM-MSC, and MSC on Genotoxicity and Cytotoxicity and Im-Munotoxicity Biomarkers

Cisplatin significantly elevated 8-OH-dG levels in sera of rats, while administration of OLE, CM-MSC, and MSC significantly decreased this biomarker level by their co-treatment with Cis in rats, indicating DNA protection against oxidative damage. LDH activity was significantly elevated in Cis-injected rats. The simultaneous administration of OLE, CM-MSC, and MSC with Cis significantly attenuated the activity of this enzyme when compared to Cis-treated group (Table 6). Serum lysozyme activity was significantly decreased in Cis-injected rats. The simultaneous administration of OLE, CM-MSC, and MSC with Cis significantly restored the lysozyme activity compared to Cis-only treated rats (*p* < 0.05). Furthermore, Cis promoted NO production (>2-fold increase compared with the control; *p* < 0.05); however, administration of OLE, CM-MSC, and MSC, with Cis significantly decreased NO levels with preference to MSC (Table 6).

### 3.6. Morphological and Phenotypic Characterization of the Isolated BM-MSCs

**Figure 1:**
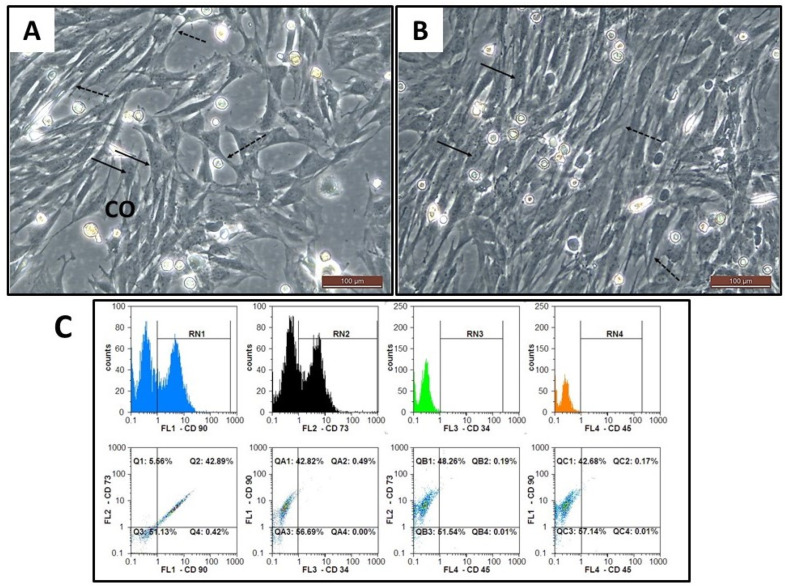
Phase-contrast photomicrographs of the isolated BM-MSCs during P0 (**A**) and P2 (**B**). The cells showed heterogeneous morphology during P0 and homogeneous fibroblast-like morphology during P2. Colonies (CO) were evident. The cells displayed granular cytoplasm with large nuclei having multiple nucleoli (Arrow) and many cytoplasmic processes (dashed arrow). (**C**): flow cytometric analysis of stem-cell marker expression on the culture MSCs during P2.

The isolated bone marrow MSCs showed physical and morphological character of stromal cells. They were plastic adherent and exhibited different cellular outlines during the primary culture (P0), ranging from polygonal to fibroblast (Figure 1A), but the fibroblast-like morphology predominated during the subsequent passages (Figure 1B). The cells appeared with granular cytoplasm, abundant nuclei, and multiple nucleoli. They possessed many cytoplasmic processes and showed a great tendency to form colonies. The phenotypic character of the cells was assessed by flowcytometry during Passage 2 (P2). Most of the cells expressed the mesenchymal stem cell marker CD 90 and 73 and lacked the hematopoietic stem cell markers CD 34 and CD 45 (Figure 1C). 

### 3.7. Red Blood Cells Micronucleus Test

**Figure 2:**
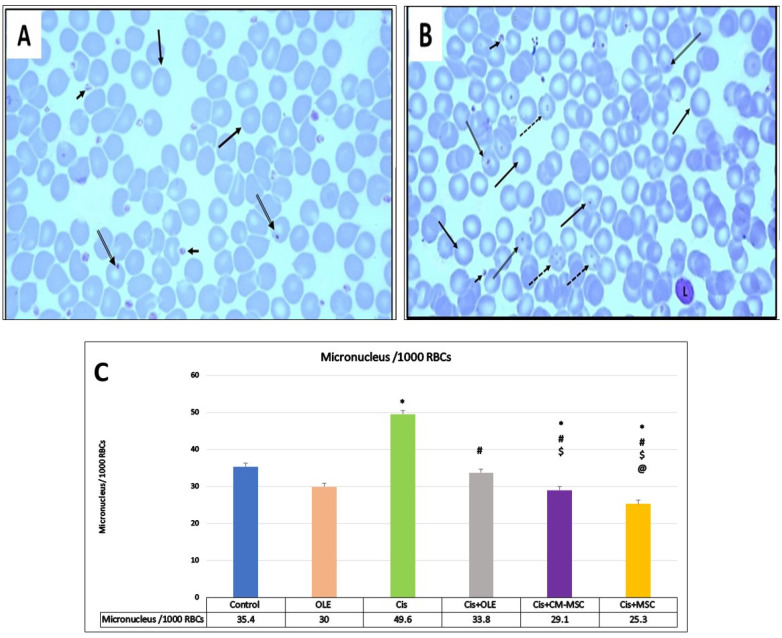
Photomicrographs of blood smear of control group (**A**) and Cis group (**B**) stained with Giemsa. Many RBCs showed normal morphology (long arrow), some were micronucleus-including (double arrow) and some echinocytes with multiple inclusion bodies (dashed arrow) were evident in the Cis group. Platelet (short arrow) and lymphocytes (L) were seen among RBCs. (**C**): A bar chart showing micronucleus frequency in 1000 cells/each group in the study. Mean ± SD was used to express the data of the experiment. Significant in comparison to control group (*), Cis group (#), Cis + OLE group ($) and Cis + CM-MSC group (@); (*p* < 0.05).

The blood smear of both control and OLE groups stained with Giemsa showed that red blood cells (RBS) were the predominant cell type in the smear. They are anucleate, non-granulated cells that are uniform in shape (biconcave discs) and size. RBCs have a central concavity that appears pale under the light microscope. Micronuclei were recognized in few uninjured cells with preserved cytoplasm in the form of spherical cytoplasmic inclusions (Figure 2A). RBCs in the Cis group showed different morphological patterns regarding the presence of inclusion bodies; many cells appeared as burr-shaped (Echinocytes) with multiple inclusion bodies, while other cells had only one micronucleus, mostly located peripherally (Figure 2B). The inclusion bodies and micronucleus-containing RBCs were less frequently seen in Cis + OLE group and were hardly seen in Cis + CM-MSC and Cis + MSC groups. Quantification of these data revealed that the frequency of RBCs with micronuclei has significantly increased in Cis group (49 ± 2.1, *p* < 0.001) when compared to control and OLE groups (35.4 ± 1.4, 30 ± 1.3, respectively), and revealed a significant decrease in their numbers in Cis + CM-MSC and Cis + MSC groups (29.1 ± 3, 25.3 ± 2.2, respectively *p* < 0.05) compared to Cis group. There was no significant difference between Cis + OLE and control group, or between the Cis + CM-MSC and Cis + MSC groups (*p* < 0.5) (Figure 2C).

### 3.8. Histopathological and Immunohistochemical Assessment of Liver Sections

**Figure 3:**
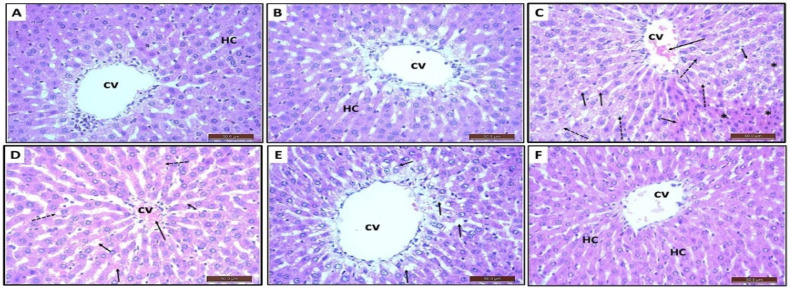
Photomicrographs of liver sections of all study groups stained with H&E. (**A**,**B**): control and OLE group showing the normal histological structure of hepatic lobules. (**C**): Cis group showed vacuolar degenerative changes in many hepatocytes (dashed arrow), congestion of central vein (long arrow), and sinusoid (short arrow). Many hepatocytes accumulate in irregular patterns disrupting the normal arrangement of hepatic cords (asterisks). (**D**–**F**): Cis + OLE, Cis + CM-MSC, and Cis + MSC groups, respectively show restoration of the normal hepatic structure in all the three groups, which is clearly seen in both Cis + CM-MSC, and Cis + MSC groups. Central vein (CV)-normally arranged hepatocyte cords (HC). ×400.

Histopathological evaluation of both control and OLE groups showed normal histological and architectural structure of the hepatic tissue. The hepatic histology showed acini and hexagonal lobules. The hepatic hexagonal lobules presented the central vein (CV) in its central position and the portal triad, which includes branches of the bile duct (BD), hepatic artery (HA), and the portal vein (PV) (Figure 3A,B). Examination of the Cis group by light microscope revealed that the hepatocytes around the central veins had dense, irregularly shaped clusters of degenerative cells with nuclei surrounded by empty vacuoles; some hepatocytes appeared as ghost cells without nuclei. There was an increase in vacuolar degeneration of hepatocytes adjacent to the central veins. Furthermore, there were massive widening and congestion in the sinusoidal spaces. Some eosinophilic hepatocytes had normal histological structures. Edema and congestion were observed in the large vessels (Figure 3C).

Examination of the Cis + OLE group showed a decrease in the number of hepatocytes with mild basophilic contents. There was a decrease in hepatocyte vacuolar degeneration in regions surrounding the central veins. Lightly stained apoptotic cells were observed besides easily identifiable normal hepatocytes. There was still some sinusoidal widening among the hepatic cords (Figure 3D).

Both Cis + CM-MSC and Cis + MSC groups showed almost restoration of normal hepatic architecture, with normally arranged hepatic cords around the central vein, with minimal residual vacuolation and congestion. There was no apparent difference between the two groups (Figure 3E,F). 

**Figure 4:**
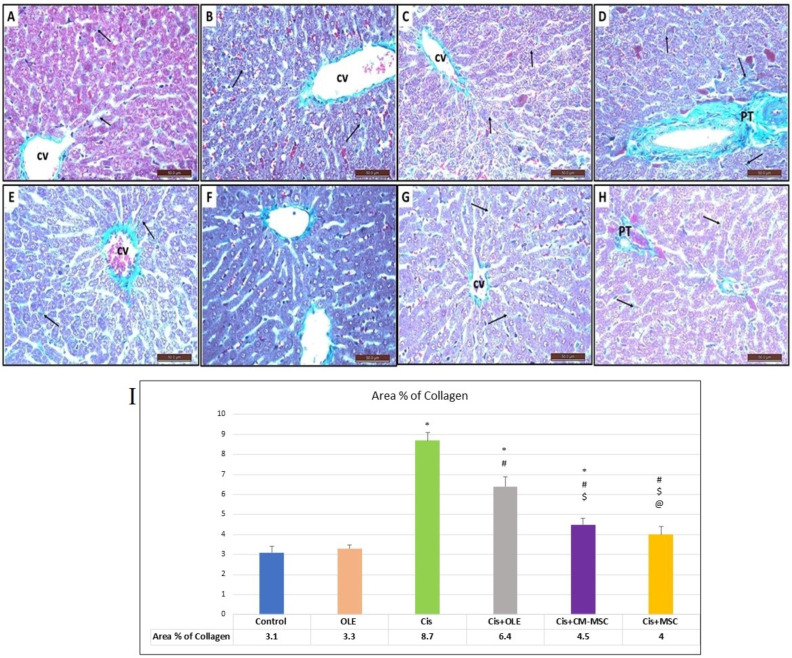
Photomicrographs of liver sections of all study groups stained with Masson Trichrome stain are showing collagen fibers distribution around the central vein (CV), portal triad (Pt), and in the connective tissue septa (arrow). (**A**,**B**): Control and OLE group, respectively. (**C**,**D**): Cis group. (**E**,**F**): Cis + OLE, Cis + CM-MSC groups, respectively. (**G**,**H**): Cis + MSC group. The expression of collagen fibers was more noticeable in the Cis group compared to the other groups. ×400. (**I**): The mean area percentage bar chart of collagen fibers in the sections of the liver. Mean ± SD was used to express the data of the experiment. 6 fields per section/rat. Significant in comparison to control group (*), Cis group (#), Cis + OLE group ($), and Cis + CM-MSC group (@); (*p* < 0.05).

By examining the Masson trichrome-stained sections of the liver specimens, minimal collagen fibers were detected around the portal triad and formed septa in between the liver lobules in both control and OLE groups (Figure 4A,B). While in Cis group, large fibrous septa formation and fibers accumulation were obviously seen around the portal triad and central veins (Figure 4C,D). In the treated groups, administration of OLE has moderately improved the hepatic architecture (Figure 4E), while great improvement was observed in the groups that received CM-MSC and MSC ((Figure 4F–H). Quantification of the area percentage of collagen fibers revealed a significant increase in the Cis-treated group in comparison to the Control group (*p* < 0.05), while the same percentage decreased significantly in all Cis + OLE, Cis + CM-MSC, and Cis + MSC groups. There was a significant difference between the Cis + OLE group and both Cis + CM-MSC and Cis + MSC groups (*p* < 0.05), but the latter two groups showed no significant difference between them (*p* < 0.5) (Figure 4I).

**Figure 5:**
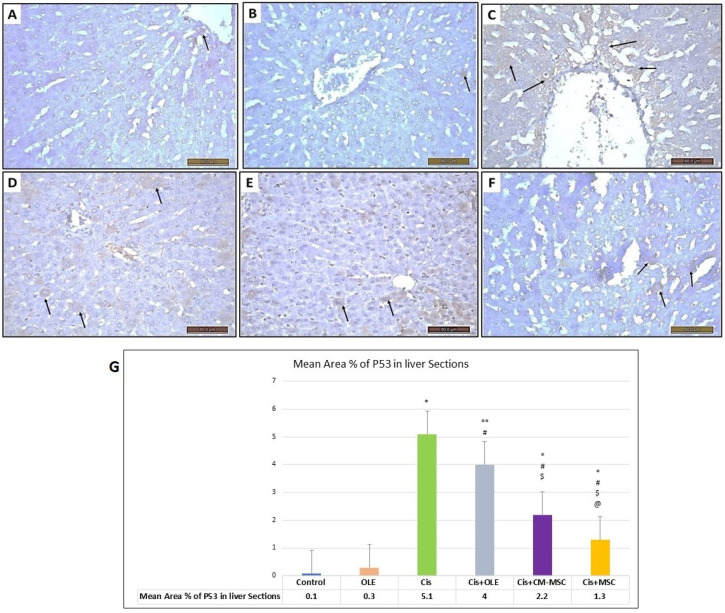
Photomicrographs of liver sections of all study groups immunohistochemically stained with anti P53 antibody. Control (**A**) and OLE (**B**) groups showed almost no expression of P53 antibody. While Cis group (**C**) showed apparent increase of its expression in some hepatocytes in comparison to Cis + OLE (**D**) and Cis + CM-MSC (**E**) groups. Minimal expression was observed in Cis + MSC group (**F**) (arrow). (**G**) The mean area percentage bar chart of P53 positively stained cells in the sections of the liver. Mean ± SEM was used to express the data of the experiment. 6 fields per section/rat. Significant in comparison to control group (*), Cis group (#), Cis + OLE group ($) and Cis + CM-MSC group (@); (*p* < 0.05).

Immunohistochemical analysis of P53 antibody expression in the liver of Cis group (Figure 5C) was significantly higher than in the control and OLE groups (*p* < 0.05) (Figure 5A,B), moderately decreased in Cis + OLE group (*p* < 0.05), and highly decreased in both Cis + CM-MSC and Cis + MSC groups compared to the Cis group (*p* < 0.05) (Figure 5D–F). A significant difference was recognized between the Cis + OLE group and both Cis + CM-MSC and Cis + MSC groups (*p* < 0.05), but the latter two groups showed no significant difference between them (Figure 5G).

### 3.9. Histopathological and Immunohistochemical Assessment of Kidney Sections

**Figure 6:**
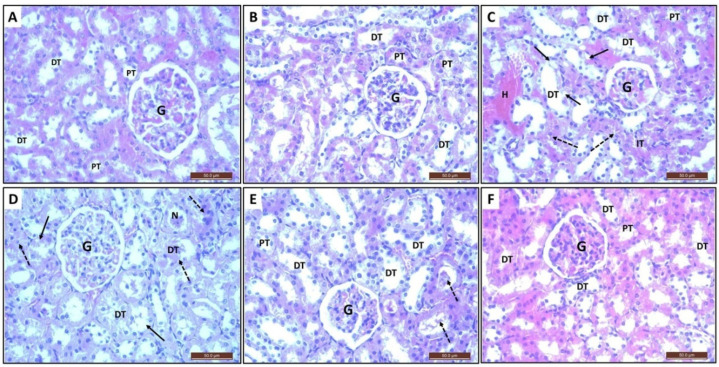
Photomicrographs of renal tissues of all study groups stained with H/E. (**A**,**B**): Control and OLE group respectively showing the normal histological structure of renal parenchyma. (**C**): Cis group showed tubular degenerative changes and glomerular shrinkage, focal hemorrhage, and congestion of blood vessels. (**D**–**F**): Cis + OLE, Cis + CM-MSC, and Cis + MSC groups, respectively. Regeneration of the epithelium of the tubules occurred in the three groups, but obviously noticed in both Cis + CM-MSC and Cis + MSC groups. G: glomerulus, DT: distal convoluted tubule, PT: proximal convoluted tubule, H: hemorrhage, IT: interstitial necrotic tissue, Arrow: vacuolation of tubular epithelium, Dashed arrow: obliterated tubular lumen with necrotic tissue.

Histopathological examination of renal tissue from control and OLE groups stained with H/E displayed the tubules and glomeruli with normal structure (Figure 6A,B). Renal sections in Cis group showed severe degenerative changes and coagulative necrosis in the tubular lining epithelium of the distal convoluted tubules (DT). Many DTs showed vacuolization of the tubular epithelial cells, tubular dilatation, and slogging of almost entire epithelium in some specimens due to desquamation of the tubular epithelium. Obliteration of some tubular lumens with necrotic tissue was also noticed. Congestion of inter-tubular blood vessels and focal hemorrhage were clearly observed. Accumulation of necrotic tissue inside the tubular lumens and in the interstitial spaces was observed (Figure 6C). Administration of either OLE, CM-MSCs, or MSCs resulted in improvement of the overall histopathological picture of the kidneys, with a better improvement observed in Cis + CM-MSC and Cis + MSC groups when compared to Cis + OLE group (Figure 6D–F).

**Figure 7:**
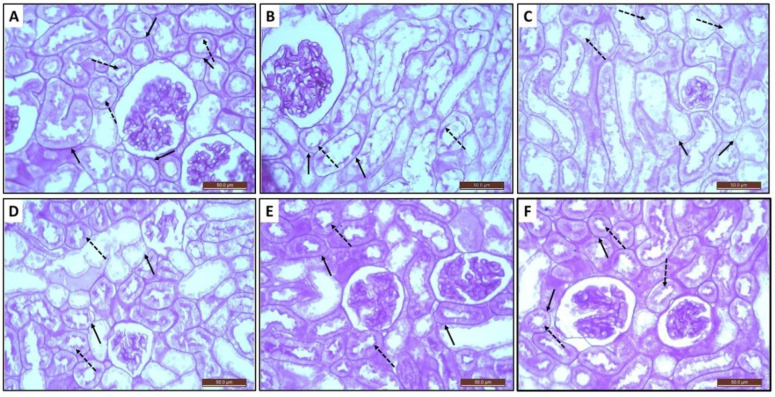
Photomicrographs of renal tissues of all study groups stained with periodic acid Schiff (PAS). (**A**,**B**): Control and OLE group showing the renal parenchyma with normal architecture as well as positive PAS material expression in the basement membrane (arrow) and the cilia of the proximal convoluted tubules lining epithelium. (**C**): Cis group displayed disorganization of the tubules and shortage of the tubular epithelium expression of positive material of PAS. (**D**–**F**): Cis + OLE, Cis + CM-MSC, and Cis + MSC groups, respectively. Regeneration of the epithelium of the tubules occurred in the three groups, but was obviously noticed within the last two groups, indicated by the brush border stained positively by PAS. Arrow: basement membrane. Dashed arrow: brush border of tubular epithelium.

Renal sections in Cis group that were stained with periodic acid Schiff (PAS) showed disintegration of the basement membrane of the tubules, remarkable thickening of the mesangial matrix of the glomeruli, as well as devastation of the epithelial cilia of the proximal convoluted tubules (Figure 7C) in comparison to both control and OLE groups (Figure 7A,B). The normal architecture of the glomeruli and tubules was restored in Cis + CM-MSC and Cis + MSC groups more than that which occurred in the Cis + OLE group (Figure 7D–F).

**Figure 8:**
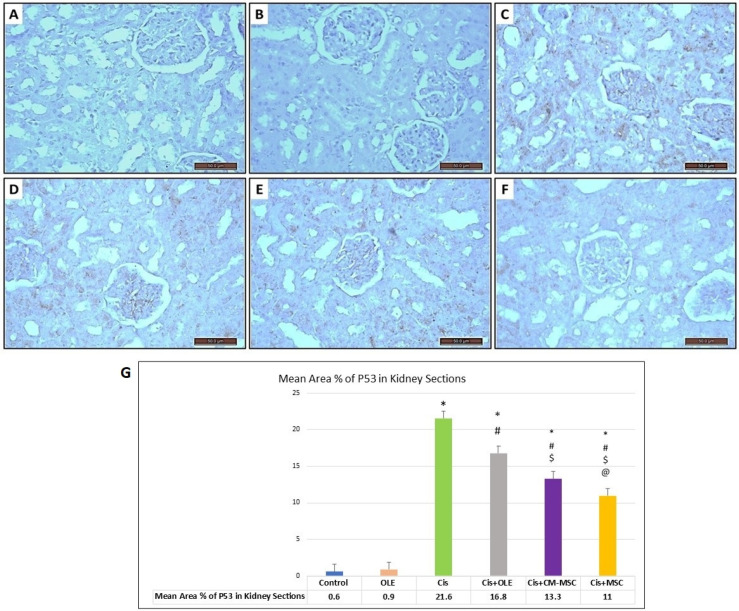
Photomicrographs of anti P53 immunohistochemically stained renal sections of all study groups. Control (**A**) and OLE (**B**) groups showed almost no expression of P53 antibody. Cis group (**C**) showed apparent increase of P53 expression in comparison to Cis + OLE (**D**) and Cis + CM-MSC (**E**) groups. The positive expression was mainly observed in the tubular epithelium and in some few glomerulus cells. Minimal expression was observed in Cis + MSC group (**F**). (**G**) The mean area percentage bar chart of P53 positively stained cells in the sections of the kidney. Mean ± SD was used to express the data of the experiment. 6 fields per section/rat. Significant in comparison to control group (*), Cis group (#), Cis + OLE group ($), and Cis + CM-MSC group (@); (*p* < 0.05).

Immunohistochemical expression of P53 antibody in renal sections appeared as brown cytoplasmic discoloration in both tubular epithelium and glomerular cells (Figure 8). The expression in the renal sections of Cis group (Figure 8C) was much higher than in the control and OLE groups (*p* < 0.05) (Figure 8A,B). P53 antibody expression decreased moderately in Cis + OLE treated rats (*p* < 0.05) and highly decreased in both Cis + CM-MSC and Cis + MSC groups compared to the Cis group (*p* < 0.05) (Figure 8D–F). A significant difference was detected between the Cis + OLE treated rats and both Cis + CM-MSC and Cis + MSC groups (*p* < 0.05), but the latter two groups showed no significant difference between them (Figure 8G).

### 3.10. Histopathological and Immunohistochemical Assessment of Thymus Sections

**Figure 9:**
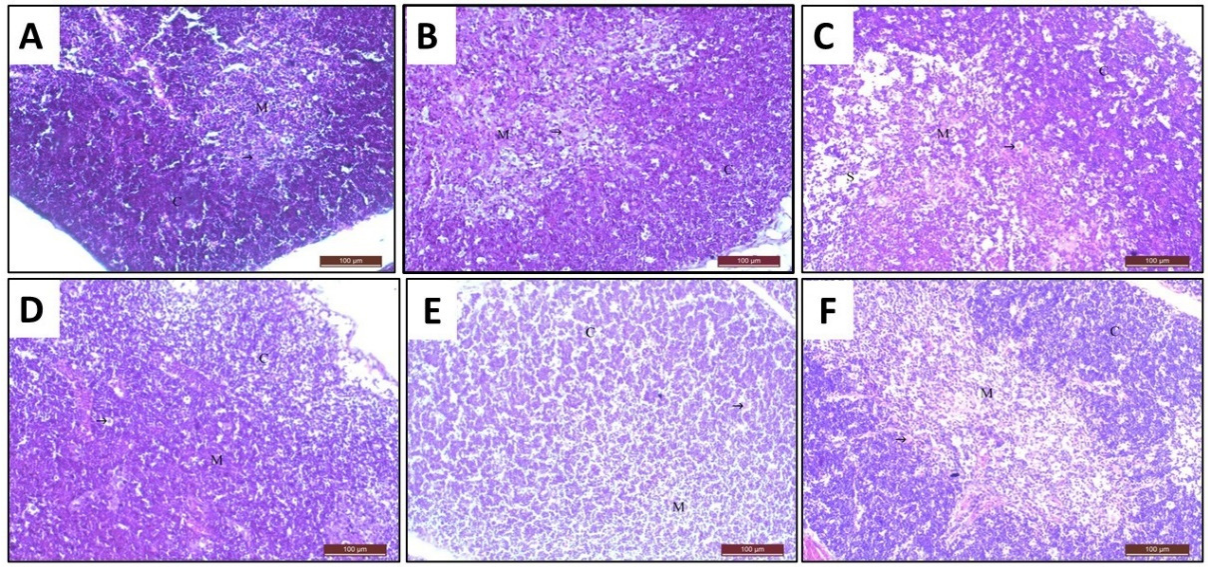
Photomicrographs of section of the thymus stained with H&E ×200 from different groups. (**A**)**:** (control group) shows normal histologic structure of the cortex (C), medulla (M), and Hassal’s corpuscles (→). There is an increased density of the lymphocytes in the cortex. (**B**): (OLE group) is similar to control group. (**C**): (Cis group) shows marked decrease in lymphocytes density in the cortex (C) and medulla (M). Also, there is an increase in Hassal’s corpuscles (→) in the medulla. There are empty spaces in the medulla (S). (**D**): (Cis + OLE group) shows restoration of normal histological structure of the thymus but less than Cis + MSC group. In addition, there are Hassal’s corpuscles (→). (**E**): (Cis + CM-MSC group) shows decreased lymphocytes density in the cortex (C) and medulla (M), but it is more similar to Cis + MSC group (**F**): (Cis + MSC group) shows restoration of normal histological structure of the thymus, but there is mild decreased lymphocyte density in the cortex (C) and medulla (M). In addition, there are Hassal’s corpuscles (→).

**Figure 10:**
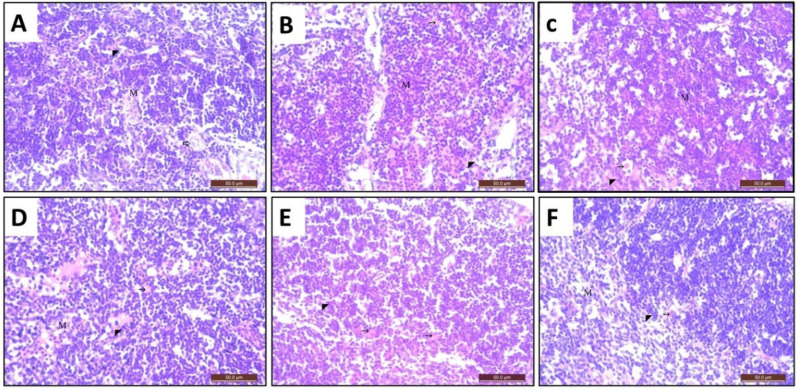
Photomicrographs of sections of the thymus stained with H/E ×400 from different groups. (**A**): (Control group) shows medulla, epithelioreticular cells (arrowhead), and Hassal’s corpuscle (→). (**B**): (OLE group) is similar to control group. (**C**): (Cis group) shows obvious spaces in the medulla due to decreased density of lymphocytes. (**D**): (Cis + OLE group) shows increased lymphocytes density more than that in Cis group. (**E**): (Cis + CM-MSC group) shows increased lymphocytes density but less than that in Cis + MSC group. (**F**): (Cis + MSC group) shows less lymphocytes density than that in control group, but more than that in Cis group.

H/E-stained sections of the thymus were examined histologically to assess the effect of the different administered treatments. Control and OLE groups showed normal structure of the thymus, which is composed of cortex and medulla. The cortex is more heavily populated with T-lymphocytes than the medulla, which appeared paler than cortex. In the medulla, Hassall’s corpuscle was identified (Figure 9A,B and Figure 10A,B). Cisplatin group showed marked decrease in lymphocytes density in the cortex and medulla. In addition, there was an increased number of Hassal’s corpuscles in the medulla. There were empty spaces in the medulla (Figure 9C and Figure 10C). Cis + OLE, Cis + CM-MSC, and Cis + MSC groups showed restoration of the normal histological structure of the thymus (Figure 9D–F and Figure 10D–F).

**Figure 11:**
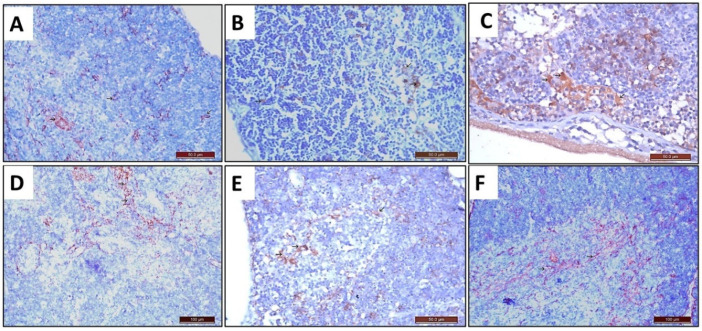
Photomicrographs of sections of the thymus stained with anticytokeratin ×400 from different groups. (**A**): (Control group) shows a positive brownish immunoreaction in some cells of the reticuloepithelial cells (→) in the cortex and medulla. (**B**): (OLE group) is similar to control group. (**C**): (Cis group) shows an obvious increase in the positive brownish immunoreaction in some cells of the reticuloepithelial cells (→) in the cortex and medulla. (**D**): (Cis + OLE group) shows a decrease in the positive brownish immunoreaction in the reticuloepithelial cells (→) in the cortex and medulla. (**E**): (Cis + CM-MSC group) shows a decrease in the positive brownish immunoreaction in the reticuloepithelial cells (→) in the cortex and medulla. (**F**): (Cis + MSCs group) shows an obvious decrease in the positive brownish immunoreaction in the reticuloepithelial cells (→) in the cortex and medulla.

Immunohistochemically stained sections with anticytokeratin of control and OLE groups showed few scattered reticuloepithelial cells in the cortex and medulla (Figure 11A,B; Table 7). The Cis-treated group revealed an obvious increase in the positive brownish immunoreaction of the reticuloepithelial cells in the cortex and medulla (Figure 11C; Table 7). However, Cis + OLE, Cis + CM-MSC, and Cis + MSC groups showed a decrease in the positive brownish immunoreaction in the reticuloepithelial cells in the cortex and medulla. Cis + MSCs group had obviously the least detected positive brownish immunoreaction (Figure 11D–F; Table 7).

### 3.11. Histopathological and Immunohistochemical Assessment of Spleen Sections

**Figure 12:**
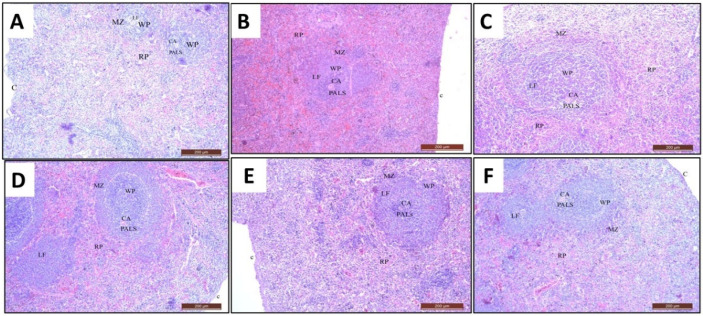
Photomicrographs of section of the spleen stained with H/E ×100 from different groups. (**A**): (Control group) shows capsule (C), white pulp (WP), and red pulp (RP). The white pulp is formed of central artery (CA), periarteriolar lymphatic sheath (PALS), lymphoid follicle (LF), and marginal zone (MZ). (**B**): (OLE group) is similar to control group. (**C**): (Cis group) shows thick capsule and thick trabeculae. There is decreased white pulp density and congested red pulp. (**D**–**F**) Cis + OLE, Cis + CM-MSCs, and Cis + MSC groups respectively show restoration of normal histological structure of the spleen.

**Figure 13:**
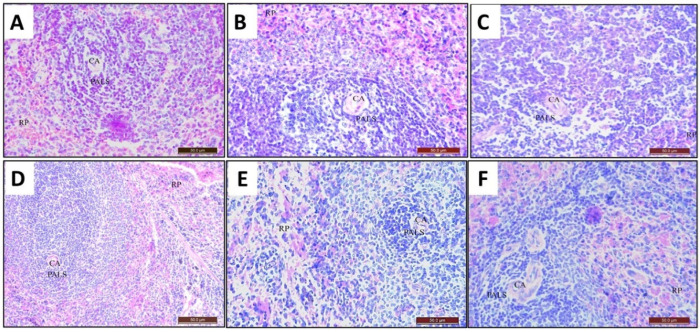
Photomicrographs of section of the spleen stained with H/E ×400 from different groups. (**A**): (Control group) shows capsule (C), white pulp (WP), and red pulp (RP). The white pulp is formed of central artery (CA), periarteriolar lymphatic sheath (PALS), lymphoid follicle (LF), and marginal zone (MZ). (**B**): (OLE group) is similar to control group. (**C**): (Cis group) shows small white pulp density and congested red pulp. (**D**): (Cis + OLE group) shows small white pulp density and congested red pulp, but better than cisplatin group. (**E**): (Cis + CM-MSC group) shows small white pulp density and congested red pulp, but better than Cis group. (**F**): (Cis + MSC group) shows restoration of normal histological structure of the spleen nearly similar to control group.

Stained sections of the spleen with H/E of control and OLE groups show that were covered by a capsule. The parenchyma is composed of white pulp and red pulp. The white pulp is formed of the central artery with periarteriolar lymphatic sheath forming lymphoid follicle (Figure 12A,B and Figure 13A,B). Cisplatin group showed decreased white pulp density and congested red pulp (Figure 12C and Figure 13C). The spleen in Cis + OLE, Cis + CM-MSC, and Cis + MSC groups regained the normal histological appearance (Figure 12D–F and Figure 13D–F).

**Figure 14:**
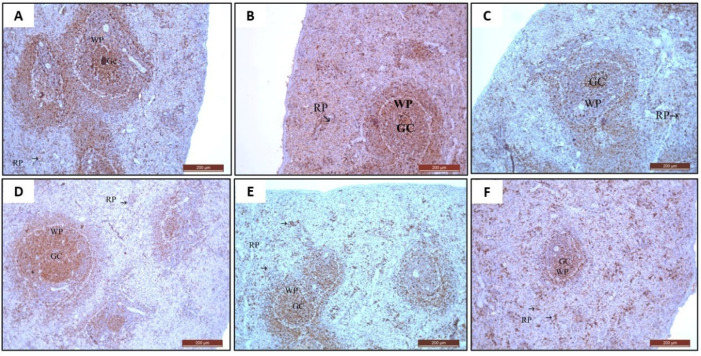
Photomicrographs of section of the spleen stained with anti-PCNA ×100 from different groups. (**A**): (Control group) shows positive brownish immunoreaction in the cells of the white pulp (WP) and in the cells of the red pulp (RP). (**B**): (OLE group) is similar to the control group. (**C**): (Cis group) shows a marked decrease in the brownish immunoreaction in the cells of the white pulp (WP) and in the cells of the red pulp (RP). (**D**): (Cis + OLE group) shows an increase in the brownish immunoreaction in the cells of white pulp (WP) and in some cells of the red pulp (RP). (**E**): (Cis + CM-MSC group) shows increased brownish immunoreaction in the cells of the white pulp and in some cells of the red pulp (RP). (**F**): (Cis + MSC group) shows increased brownish immunoreaction in the cells of the white pulp (WP) and in some cells of the red pulp (RP).

Immunohistochemically stained sections with anti-PCNA in both control and OLE groups revealed positive brownish immunoreaction in some cells of the red pulp and negative immunoreaction in the cells of the white pulp (Figure 14A,B; Table 8). However, PCNA was immunopositive in all subgroups treated by cisplatin; there was a difference of positivity level among the groups of animals in the white pulp. Cisplatin showed a marked decrease in the brownish immunoreaction in the cells of white pulp and in some cells of the red pulp (Figure 14C; Table 8). Cis + OLE, Cis + CM-MSC, and Cis + MSC groups showed brownish immunoreaction in the cells of white pulp and in some cells red pulp more than cisplatin group. PCNA indicated cellular proliferation (Figure 14D–F; Table 8).

### 3.12. Quantitative Real-Time Polymerase Chain Reaction (RT-PCR) Analysis of Bcl-2, Bax, and Caspase-3 Genes

**Figure 15:**
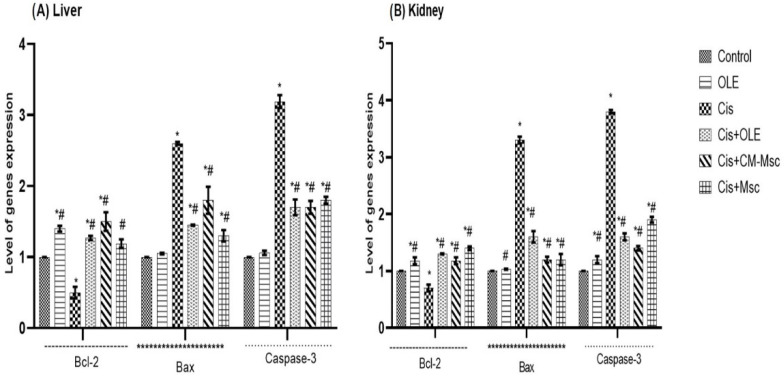
Bcl-2, Bax, and caspase-3 genes expression level (**A**): liver tissues, (**B**): kidney tissues. Values are expressed as mean ± SD. One way ANOVA was used to analyze the data. Statistically significant compared to control group (*) and cisplatin group (#); *p* < 0.05.

The Bcl-2 gene expression levels in the liver and kidney tissues were significantly lower in the Cis-treated group than in the control group (Figure 15A,B). Treatment with OLE, CM-MSC, and MSC resulted in restoration of Bcl-2 expression levels in both the liver and kidney compared to the Cis-treated group. On the other hand, both Bax and caspase-3 genes showed significantly higher expression levels in the liver and kidneys in Cis-treated group than in the control group. Treatment with OLE, CM-MSC, and MSC significantly lowered the liver and kidney Bax, and caspase-3 gene expression compared to the Cis-treated group (Figure 15A,B).

### 3.13. Polymerase Chain Reaction Analysis of Sry Gene

**Figure 16:**
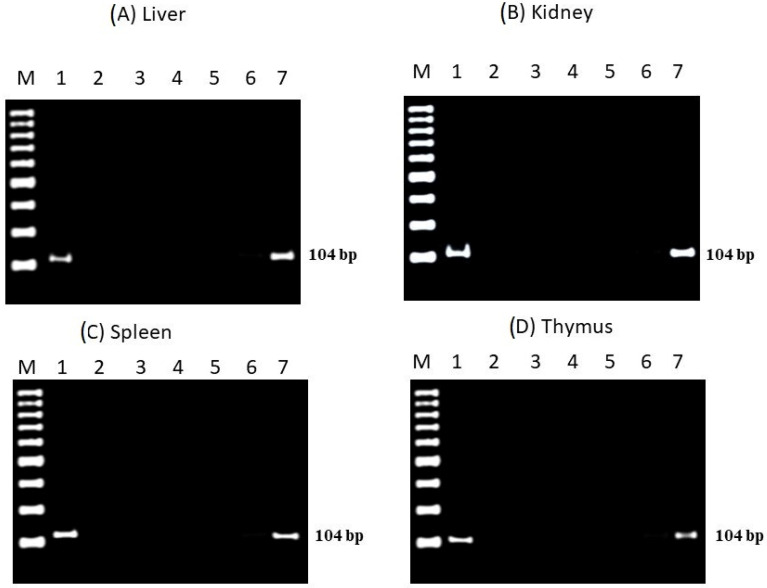
PCR for sry gene in the liver (**A**), kidney (**B**), spleen (**C**), and thymus gland (**D**) of rats; M: 100 bp DNA maker; sry: 104 bp, lane 1: Control positive; lane 2: Control negative; lane 3: OLE; lane 4: Cis; lane 5: Cis + OLE; lane 6: Cis + CM-MSC; lane 7: Cis + MSC.

After 3 weeks of MSCs transplantation, real-time PCR assay revealed that sry gene was detected and tracked in DNA content of liver, kidney, thymus, and spleen of MSCs-treated females, whereas all other groups showed no PCR product of sry gene (Figure 16).

## 4. Discussion

Cisplatin is a widely used chemotherapeutic agent. However, its deleterious effects on body systems are a major concern that limits its use [9]. The results of the present study compared the ameliorative effect of OLE, CM-MSC, and BM-MSC on Cis-induced hepato-renal toxicity, immunotoxicity, and cyto-genotoxicity in rats, with preference to MSCs.

Various studies have linked Cis-induced hepato-renal damage to the oxidative stress, and acknowledged the promising ameliorative action of substances with antioxidant activity against Cis-induced hepato-renal toxicity [11,12,13,14,15,16,17,76,77]. In our study, we found that Cis elevated hepatic and renal lipid peroxidation product, MDA, and at the same time, it reduced the levels of antioxidant enzymes (CAT, SOD), GSH, and TAC. When Cis enters the cell, it becomes detoxified through conjugation with GSH [78]. Continuous exposure to Cis or exposure to large doses of Cis depletes GSH stores and makes Cis readily available to bind to both nuclear and mitochondrial DNA, resulting in cell death. When Cis binds to mitochondrial DNA, it forms adducts leading to impairment of mitochondrial functions and ROS production, promoting cell death and further cellular damage [22,79]. Furthermore, it reduces catalase enzyme, leading to an increase in ROS production and lipid peroxidation [76].

ROS stimulate tumor suppressor p53 phosphorylation, resulting in transcription of pro-apoptotic proteins and activation of apoptotic pathways [22]. In agreement with other studies [13,14,15,17], we detected increased expression of P53 in the livers and kidneys of Cis-treated rats. Both BAX and Bcl-2 are pro- and anti-apoptotic molecules that control Cis-induced apoptosis. In agreement with other studies [11,12,13,14,15,22,80,81], we detected increased BAX and caspase-3 and decreased Bcl-2 expression in hepatic and renal tissues of Cis-treated rats. Bcl-2 is an anti-apoptotic protein, encoded by the Bcl-2 proto-oncogene, and is one of the molecular members of the Bcl-2 family of cell survival factors. It is present on the mitochondrial outer membrane and blocks the release of cytochrome C from the mitochondria, preventing apoptosis. Its anti-apoptotic effect results from its ability to counter the apoptotic protein BAX, and hence stabilizes the integrity of mitochondrial membrane. Under oxidative stress conditions, BAX promotes mitochondrial membrane permeability, and leads to leak of cytochrome C and ROS into the cytoplasm from the mitochondria, ending up with activation of caspase 3, the regulator of apoptosis [82]. Apoptosis was confirmed histologically in hepatic and renal tissues of rats injected with Cis in the present study.

The present study revealed elevated levels of hepatic and renal inflammatory markers IL-1β, IL-6, and TNF-α, confirming the role of Cis-induced inflammation in hepatic and renal damage, in agreement with other studies [11,14,15,16,17]. Cis-induced cellular damage induces the release of molecular pattern molecules as box-1 [83] that activates toll-like receptors (TLRs). TLRs activate nuclear factor kappa B (NF-kB), which promotes tumor necrosis factor alpha (TNF-α) production that leads to production of other inflammatory cytokines [13,14,15,16,17,22]. Furthermore, TNF-α was found to be responsible for apoptosis, and its reduction leads to apoptosis amelioration [11].

The previously integrated mechanisms emphasize the role of substances with antioxidant, anti-inflammatory, and anti-apoptotic actions in ameliorating Cis-induced organ damage. The present study revealed that OLE improved Cis-induced liver and kidney damage and enhanced their functions. In the same context, OLE provided liver protection against fluoxetine [48] and renal protection against Cis [84] and amikacin [85]. In addition, it ameliorated hepato-renal toxicity induced by doxorubicin [86].

OLE is a natural product derived from olive trees. It contains mainly oleuropein and other phenolic compounds, flavonoids, and tocopherol [41]. The present study revealed that OLE ameliorated Cis-induced hepatic and renal damage, and improved their functions through its antioxidant, anti-apoptotic, and anti-inflammatory activity. In congruence with our findings, many studies reported the capability of OLE to reduce the oxidative stress, exert anti-apoptotic action, and suppress the levels of inflammatory markers in hepatic and renal tissue [48,84,87].

Olive phenolic compounds decreased oxidative stress, improved antioxidant enzymes levels, and exerted anti-apoptotic activity through decreasing the expression of P53 and increasing the expression of Bcl-2 proteins in hepatic and renal tissues of deltamethrin-treated rats [45]. Oleuropein, the main phytochemical component in OLE, also exerted antioxidant activity in renal tissue of Cis-injected rats [44]. However, studies have proved that OLE exerted greater protective, antioxidant, and even regenerative effects than oleuropein alone in mice suffering from Cis-induced reproductive toxicity [88]. Researchers attributed the higher antioxidant activity of OLE to the synergism between the flavonoids, substituted phenols, and oleuropeosides present in olive leaves rich in oleuropein [47].

In the current study, MSCs were able to home into livers, kidneys, thymus, and spleen of Cis-treated rats. Both MSCs and their conditioned media proved to be effective against Cis damaging effects; however, there is a preference for MSC over CM-MSC. They were able to improve hepatic and renal structure and function and reduce Cis-induced oxidative stress, inflammation, and apoptosis. The beneficial effects of stem cells might occur through differentiation-independent pathways that include enhanced cellular proliferation and survival and anti-inflammatory, anti-apoptotic, immunomodulatory, and angiogenic action [89].

Stem cells release microvesicles that are categorized into shedding vesicles and exosomes. Microvesicles convey lipids, proteins, and nucleic material to recipient cells; therefore, they are considered important paracrine mediators, and they are involved in signaling between stem and differentiated cells. They may provoke development regulation, cell differentiation, and regeneration. Through their paracrine action, stem cells have been reported to interact with the surrounding cells, leading to enhancement of stem cell division and further recruitment of stem cells from stem cell niches. This action improves stem-cell-derived tissue repair [90].

Our results revealed the healing and renoprotective powers of both BM-MSCs and their conditioned media in Cis-treated rats. MSCs show promising results in kidney repair, due to the mesenchymal embryonic source of nephrons besides the crucial importance of stromal cells for signaling and paracrine action, promoting the differentiation of both nephrons and collecting ducts [91]. Our results are in line with studies that proved that BM-MSCs can fuse with original renal cells and replace 50% of the injured proximal tubular epithelium after transplantation [92]. Angiogenesis action and differentiation potential of BM-MSCs were evident after their homing to injured glomerular endothelium and its reconstruction, in addition to participation in regeneration of the glomerular microvasculature [93] or by decreasing inflammatory and apoptotic effects in Cis-treated rats.

Infusion of conditioned media in the current research was very promising for protecting renal tissue against Cis toxicity. Besides their content of growth factors and cytokines, conditioned media of many cultured types of stem cells also contain exosomes that can mediate side-talks between cells [94]. MSC conditioned media could resist Cis-induced renal damage through their antioxidant, anti-inflammatory, and anti-apoptotic actions, besides autophagy stimulation [38,95,96].

We found that stem cells and their conditioned media proved to restore the liver structure and function. The transplanted MSCs act by differentiation into functional hepatocytes. In addition, through their paracrine action, they release an array of cytokines and growth factors that are able to ameliorate liver tissue apoptosis, inflammation, and fibrosis, and furthermore, they can improve liver cell function. Various studies revealed the in vitro differentiation capability of MSCs along the hepatogenic lineage [97]. A previous study reported that native as well as MSCs-derived liver cells could be recruited and were able to differentiate into effective liver cells inside recipients’ livers [98]. Exosomes derived from MSCs had the ability to suppress carbon tetrachloride-induced hepatic damage through their antioxidant functions [99].

Systemic infusion of CM-MSC was proved to significantly enhance liver cell survival and ameliorate liver damage [100]. Furthermore, they were reported to significantly decrease liver cell apoptosis and support liver regeneration. The high affinity of MSCs to home into injured tissues, beyond their sustained delivery of trophic signals, give them superiority over CM-MSCs. Nevertheless, long-term survivability is weak, and unwanted pathways of differentiation should be considered [101].

Cis-induced immune suppression was reported in the literature. It was attributed to the action of Cis as an alkylating agent; as it binds to DNA, forming cross-links, and inhibits cell replication, in addition to induction of ROS and apoptosis of bone marrow cells [102] and spleen [103]. The present study revealed Cis-induced alteration of the histological structure of the spleen and thymus, organs essential for adaptive immunity. This finding is in line with other studies, as Cis was proved to cause depletion of the lymphoid tissue, apoptosis, and hemosiderosis in the spleen [102,103,104,105,106].

We detected the ability of OLE to protect against Cis-induced lymphocytotoxicity as evidenced by protecting the lymphoid organs (spleen and thymus) from Cis-induced structural damage and lymphoid depletion. Our results are in agreement with Khalil et al., who proved the ability of EVOO, through its antioxidant activity, to improve the histological structure of the spleen and increase T and B lymphocytes production [107]. We believe that OLE protected against Cis-induced lymphoid tissue destruction through its antioxidant and anti-apoptotic activity. In the same context, we found that both BM-MSCs and their conditioned media migrated into the spleen and thymus and improved the histological structure of the thymus and spleen. Our results agree with Wang et al. who documented the ability of BM-MSCs, through their antioxidant activity, to improve the structure and function of thymus and spleen in aging rat model. Furthermore, MSCs increased proliferation and DNA replication of the splenocytes and thymocytes as proved with increased PCNA expression in both lymphoid organs [108]. PCNA plays an important role in DNA replication, and its expression is an indicator of cellular proliferation [109]. In the present study, we detected that OLE, MSCs, and their conditioned media increased PCNA expression in the spleen, which might indicate their ability to enhance cellular proliferation, or indicate their ability to protect against DNA damage that hinders cellular proliferation. Studies have clarified that MSCs, through their paracrine mediators, can either promote or suppress the immune response according to the micro-environmental inflammatory conditions to control tissue repair and establish homeostasis. For example, in severe inflammatory conditions, MSCs can stimulate the expansion of T regulatory and B regulatory cells in the lymphoid tissue [110,111]; however, we did not identify the specific type of proliferating cells in the lymphoid organs in rats exposed to MSCs or their conditioned media.

In the present study, we assessed thymus regeneration using anti-cytokeratin immune staining. This stain detects cytokeratin (protein) filaments present in thymus epithelial cells (TECs). TECs are nursing epithelial reticular cells (ERCs) existing in both the cortex and medulla of the thymus, and their main function is to govern, nurse, and promote differentiation of thymocytes [112]. The increased cytokeratin expression reflects regeneration of the progenitors’ cells and their tendency to compensate for ERCs lost via apoptosis [113], which explains our finding of increased cytokeration expression in compensation for Cis-induced apoptosis in thymic tissue. In agreement with our study, cytokeratin was highly expressed during thymus regeneration after involution from acute cyclophosphamide toxicity, indicating an increased number of ERCs to compensate for thymic tissue damage. However, cytokeratin expression decreased over time and returned to normal levels within two weeks, indicating complete thymus regeneration [112]. OLE, MSCs, and their conditioned media decreased cytokeratin expression in the thymus of Cis-treated rats. We think that results might reflect the ability of the used therapeutic modalities to protect against and to lessen Cis-induced apoptosis of thymic tissue, or might reflect their ability to rapidly improve and complete thymus regeneration, which possibly decreased the further need for compensatory mechanisms.

We further investigated the ability of OLE, MSCs, and their conditioned media to enhance the immune system function through assessment of lysozyme activity. We detected that those therapeutic modalities improve Cis induced down-regulation of lysozyme activity. In line with other studies [17,114], Cis increased the production of NO in the present study. ROS are associated with macrophages and leukocyte migration to inflammatory sites. Those cells release inflammatory cytokines that lead to overexpression of inducible nitric acid oxidase (iNOS), leading to NO overproduction and resulting in nitrosative stress [43]. Peroxynitrite resulting from the reaction of NO with superoxide radical is responsible for the decrease in lysozyme activity and increases its susceptibility to proteasomal degradation [115]. Khalil et al. reported that phenolic compounds present in EVOO ameliorated the hexavalent chromium-induced immunotoxicity and enhanced lysozyme activity in rats through their antioxidant activity and their ability to reduce NO production [107]. This encourages us to assume that OLE, MSCs, and their conditioned media enhanced lysozymes activity through their antioxidant activity.

Genotoxicity and DNA damage are important mechanisms involved in Cis-induced organ damage. DNA damage results from the binding of Cis to DNA purine bases, forming adducts, leading to inhibition of DNA replication and transcription, ending up with apoptosis [116,117]. In line with other studies [1,9,118], Cis-treated rats in the present study showed increased numbers of micronucleated cells and inclusion bodied, indicating cyto-genotoxicity. A possible relation exists between micronuclei formation and DNA breakage into small fragments, as those small fragments might form multiple nuclei [107]. Studies reported that Cis-induced oxidative stress and ROS have deleterious genotoxic effects [10,17,44].

In the present study, OLE, MSCs, and their conditioned media ameliorated Cis-induced DNA damage. They were able to reduce inclusion bodies and micronuclei formation. Furthermore, they decreased production of 8-OH-dG, the marker of DNA damage. Studies reported that OLE decreased Cis-induced DNA damage in rats [84]. It was able to decrease estradiol and diethylstilbestrol induced DNA damage in human peripheral blood cells through its antioxidant and radical scavenging properties [119]. It had the ability to abolish cadmium-induced micronuclei formation [120]. In the same context, oleuropein, the principal component in OLE, ameliorated Cis-induced genotoxic effect and decreased 8-OH-dG levels through its antioxidant activity [44,121]. The ability of MSCs and their conditioned media to ameliorate Cis-induced genotoxicity in the present study might be attributable to the paracrine factors of MSCs. The present study, in line with other studies [32], confirmed the antioxidant, anti-inflammatory, and anti-apoptotic role of BM-MSCs. In the same context, MSC-derived exosomes provided resistance against cis-induced oxidative stress and decreased the oxidative DNA marker 8-OH-dG [38]. BM-MSCs were found to attenuate DNA damage in myocardial cells through the release of several cytokines and growth factors that promote angiogenesis and mitigate inflammation and apoptosis. Furthermore, they were able to recruit proteins that repair DNA damage including single- and double-strand breaks [122].

OLE, MSCs, and their conditioned media provided protection against cellular damage as evidenced by reducing LDH enzyme levels in Cis-treated rats. Increased LDH enzyme levels in serum and tissues occur due to cell necrosis and death. When ROS-induced lipid peroxidation of the cell membrane occurs, it leads to cell membrane disintegration with subsequent leakage of LDH out from the cell into the circulation [107]. The polyphenolic compounds in EVOO exerted protective effects against cellular damage as evidenced by decreased LDH levels through their antioxidant activity [107]. The protective powers of the used OLE, BM-MSCs, and their conditioned media might be attributed to their antioxidant and anti-apoptotic activity.

**Scheme 2:**
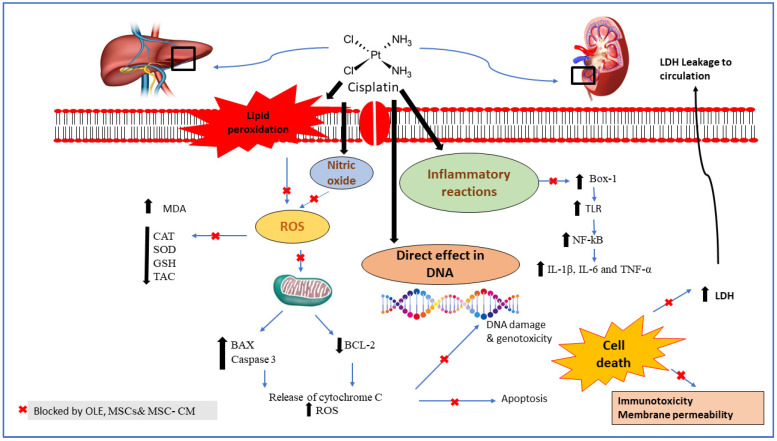
A proposed schematic diagram representing the activation of ROS, inflammatory and apoptosis signaling proteins by Cis and the role of OLE, BM-MSC, and CM-MSC to intervene in this effect in the hepatic and renal tissue. Cis: cisplatin; OLE: olive leaf extract; BM-MSC: bone-marrow-derived mesenchymal stem cells; CM-MSC: conditioned media of mesenchymal stem cells; ROS: reactive oxygen species; GSH: glutathione; MDA: malondialdehyde; CAT: catalase; SOD: superoxide dismutase; TAC: total antioxidant capacity; IL-6 and IL-1β: interleukins 6 and 1β; TNF: tumor necrosis factor; LDH: lactate dehydrogenase enzyme.

A schematic diagram representing the activation of ROS, inflammatory, and apoptosis signaling proteins by Cis and the role of OLE, BM-MSC, and CM-MSC to intervene in this effect in the hepatic and renal tissue were summarized (Scheme 2).

## 5. Conclusions

The present study succeeded in providing structural and functional evidence for the ameliorative effect of OLE, MSCs and their conditioned media against Cis-induced hepatotoxicity, nephrotoxicity, immunotoxicity and genotoxicity. Our experimental results may ascertain that OLE, MSCs and their conditioned media might modulate hepatic and renal injury through attenuation of oxidative stress, and through the subsequent crosstalk between the inflammatory and oxidative stress-mediated apoptosis cascades, with preference to MSCs. MSCs’ better results might be attributed to their sustained and continued delivery of paracrine mediators, in addition to their trans-differentiation ability. Therefore, the present study may open a new scenario for the clinical usefulness of adding OLE, MSCs or their media to chemotherapy-based treatment of cancer patients, to enhance treatment efficacy, and to ameliorate the adverse effects that might cause treatment discontinuation.

## Figures and Tables

**Table 1:** Primers sequence for Bcl-2, Bax, caspase-3 and β-actin.

Gene	Primer Sequence
Bcl-2	Forward: 5′-GGATGACTTCTCTCGTCGCTACCGT-3′
	Reverse: 5′-ATCCCTGAAGAGTTCCTCCACCAC-3′
Bax	Forward: 5′-CCAGGACGCATCCACCAAGAAGC-3′
	Reverse: 5′-TGCCACACGGAAGAAGACCTCTCG-3′
Caspase-3	Forward: 5′-GCAGCAGCCTCAAATTGTTGACTA-3′
	Reverse: 5′-TGCTCCGGCTCAAACCATC-3′
β-actin	Forward: 5′-TCCTCCTGAGCGCAAGTACTCT-3′
	Reverse: 5′-GCTCAGTAACAGTCCGCCTAGAA-3′

**Table 2:** Effects of olive leaf extract (OLE), mesenchymal stem cells (MSC), CM-MSC, cisplatin and their combination, on body, liver, and renal weight of rats.

Parameters	Control	OLE	Cis	Cis + OLE	Cis + CM-MSC	Cis + MSC
Initial body weight(g)	206.33 ± 7.66	208.00 ± 7.07	204.50 ± 5.21	205.17 ± 5.23	206.67 ± 6.65	205.67 ± 5.28
Final body weight (g)	226.33 ± 8.52	232.17 ± 9.35	194.17 ± 12.83 ^ab^	212.50 ± 19.10 ^c^	218.67 ± 13.78 ^c^	225.00 ± 10.64 ^c^
RBW	109.83 ± 6.10	111.68 ± 4.66	94.93 ± 5.46 ^ab^	103.62 ± 9.39 ^c^	105.78 ± 4.99 ^c^	109.51 ± 6.85 ^c^
Liver weight (g)	7.22 ± 0.48	7.32 ± 0.40	5.72 ± 0.59 ^ab^	6.37 ± 0.61 ^c^	6.58 ± 0.40 ^c^	6.90 ± 0.53 ^c^
RLW	3.20 ± 0.28	3.16 ± 0.21	2.96 ± 0.43 ^ab^	3.0 ± 0.11 ^c^	3.03 ± 0.36 ^c^	3.07 ± 0.29 ^c^
Kidney weight (g)	0.69 ± 0.08	0.68 ± 0.08	0.88 ± 0.07 ^ab^	0.78 ± 0.05 ^c^	0.74 ± 0.08 ^c^	0.70 ± 0.10 ^c^
RKW	0.30 ± 0.03	0.29 ± 0.04	0.45 ± 0.06 ^ab^	0.37 ± 0.04 ^c^	0.34 ± 0.05 ^c^	0.31 ± 0.05 ^c^

Data are mean ± standard deviation. OLE: olive leaves extract, Cis: cisplatin, Cis + OLE: cisplatin+ olive leaves extract. Cis + CM-MSC: cisplatin + conditioned media mesenchymal stem cells; Cis + MSC: cisplatin+ mesenchymal stem cells. RBW: relative body weight; RLW: relative liver weight; RKW: relative kidney weight. ^a^: significant compared to the control group; ^b^: significant compared to OLE group; ^c^: significant compared to Cis group. *n* = 6/group.

**Table 3:** Effects of olive leaf extract (OLE), mesenchymal stem cells (MSC), CM-MSC, cisplatin and their combination on liver and renal functions parameters of rats.

Parameters	Control	OLE	Cis	Cis + OLE	Cis + CM-MSC	Cis + MSC
Liver						
ALT (U/L)	29.50 ± 3.94	29.17 ± 3.60	142.17 ± 7.05 ^ab^	70.67 ± 5.47 ^abc^	66.33 ± 4.93 ^c^	48.33 ± 3.83 ^c^
AST(U/L)	30.90 ± 4.06	33.17 ± 3.71	132.50 ± 3.94 ^ab^	77.00 ± 4.98 ^abc^	57.33 ± 4.46 ^c^	50.67 ± 5.28 ^c^
ALP(U/L)	147.50 ± 5.24	153.67 ± 7.94	198.67 ± 5.28 ^ab^	179.83 ± 4.26 ^abc^	164.17 ± 2.64 ^c^	154.33 ± 6.28 ^c^
TP(g/dL)	6.62 ± 0.71	6.70 ± 0.69	4.45 ± 0.46 ^ab^	5.17 ± 0.26 ^abc^	5.63 ± 0.32 ^c^	6.10 ± 0.33 ^c^
Albumin (g/dL)	4.43 ± 0.37	4.30 ± 0.41	2.83 ± 0.34 ^ab^	3.10 ± 0.17 ^abc^	3.37 ± 0.39 ^c^	3.98 ± 0.22 ^c^
Total Bilirubin (mg/dL)	0.29 ± 0.04	0.24 ± 0.03	0.84 ± 0.08 ^ab^	0.56 ± 0.04 ^abc^	0.47 ± 0.09 ^c^	0.33 ± 0.05 ^c^
Kidney						
Urea(mg/dL)	27.38 ± 3.50	26.83 ± 3.66	45.17 ± 4.54 ^ab^	35.50 ± 4.64 ^abc^	31.33 ± 5.92 ^c^	29.50 ± 5.24 ^c^
Creatinine (mg/dL)	0.75 ± 0.04	0.74 ± 0.04	1.04 ± 0.18 ^ab^	0.85 ± 0.08 ^abc^	0.81 ± 0.04 ^c^	0.77 ± 0.08 ^c^

Data are mean ± standard deviation. OLE: olive leaves extract, Cis: cisplatin, Cis + OLE: cisplatin + olive leaves extract. Cis + CM-MSC: cisplatin + conditioned media mesenchymal stem cells; Cis + MSC: cisplatin+ mesenchymal stem cells. ALT: alanine transaminase; AST: aspartate transaminase, ALP: alkaline phosphatase. TP: total protein. ^a^: significant compared to the control group; ^b^: significant compared to OLE group; ^c^: significant compared to Cis group. *n* = 6/group.

**Table 4:** Effects of olive leaf extract (OLE), mesenchymal stem cells (MSC), CM-MSC, cisplatin, and their combination on liver and renal oxidative markers of rats.

Parameters	Control	OLE	Cis	Cis + OLE	Cis + CM-MSC	Cis + MSC
Liver						
CAT	348.00 ± 20.45	351.00 ± 20.69	210.50 ± 14.52 ^ab^	295.33 ± 12.48 ^abc^	298.17 ± 10.28 ^c^	309.83 ± 8.54 ^c^
SOD	288.17 ± 6.21	296.67 ± 3.33	227.17 ± 7.36 ^ab^	258.50 ± 7.89 ^abc^	264.67 ± 4.80 ^c^	284.33 ± 5.32 ^c^
GSH	6.51 ± 0.29	6.61 ± 0.38	4.32 ± 0.20 ^ab^	5.21 ± 0.48 ^abc^	5.42 ± 0.26 ^c^	6.00 ± 0.45 ^c^
TAC	88.33 ± 6.59	90.17 ± 5.88	37.50 ± 8.89 ^ab^	65.67 ± 6.53 ^abc^	77.67 ± 9.52 ^c^	83.33 ± 8.62 ^c^
MDA	49.00 ± 4.60	46.17 ± 4.45	86.17 ± 4.79 ^ab^	65.83 ± 5.74 ^abc^	62.83 ± 3.19 ^c^	53.83 ± 3.54 ^c^
Renal						
CAT	341.50 ± 17.01	343.17 ± 15.16	214.00 ± 11.10 ^ab^	299.00 ± 7.85 ^abc^	321.67 ± 16.38 ^c^	338.83 ± 10.87 ^c^
SOD	286.00 ± 6.51	283.33 ± 6.15	234.83 ± 7.81 ^ab^	254.67 ± 11.20 ^abc^	264.33 ± 7.00 ^c^	281.67 ± 7.92 ^c^
GSH	6.78 ± 0.32	6.67 ± 0.49	4.18 ± 0.33 ^ab^	5.17 ± 0.42 ^abc^	5.65 ± 0.45 ^c^	6.48 ± 0.34 ^c^
TAC	87.5 ± 6.19	85.7 ± 4.18	39.8 ± 4.49 ^ab^	66.5 ± 5.43 ^abc^	72.8 ± 6.11 ^c^	82.2 ± 3.76 ^c^
MDA	46.33 ± 5.50	47.67 ± 5.01	82.67 ± 6.25 ^ab^	56.50 ± 5.13 ^abc^	49.50 ± 4.04 ^c^	48.50 ± 4.55 ^c^

Data are mean ± standard deviation. OLE: olive leaves extract, Cis: cisplatin, Cis + OLE: cisplatin + olive leaves extract. Cis + CM-MSC: cisplatin + conditioned media mesenchymal stem cells; Cis + MSC: cisplatin+ mesenchymal stem cells. CAT: Catalase (U/gm tissue), SOD: superoxide dismutase (U/gm tissue); GSH: glutathione (mmol/gm tissue); TAC: total antioxidant capacity (U/mg); MDA: malondialdehyde (mmol/g tissue). ^a^: significant compared to the control group; ^b^: significant compared to OLE group; ^c^: significant compared to Cis group. *n* = 6/group.

**Table 5:** Effects of olive leaf extract (OLE), mesenchymal stem cells (MSC), CM-MSC, cisplatin, and their combination on the liver and renal levels of inflammatory markers of rats.

Parameters	Control	OLE	Cis	Cis + OLE	Cis + CM-MSC	Cis + MSC
Liver						
IL-1β	11.05 ± 0.34	11.08 ± 0.21	26.33 ± 0.47 ^ab^	18.78 ± 0.56 ^abc^	17.07 ± 0.62 ^c^	12.72 ± 0.57 ^c^
IL-6	110.83 ± 2.64	112.33 ± 4.23	176.50 ± 5.43 ^ab^	129.17 ± 6.62 ^abc^	121.50 ± 6.47 ^c^	118.50 ± 5.05 ^c^
TNF-α	2562.00 ± 22.30	2552.00 ± 16.70	3457.33 ± 14.47 ^ab^	2975.67 ± 17.73 ^abc^	2764.33 ± 33.18 ^c^	2602.17 ± 22.68 ^c^
Renal						
IL-1β	13.77 ± 1.00	14.07 ± 0.81	30.6 ± 0.75 ^ab^	20.15 ± 1.01 ^abc^	18.0 ± 1.11 ^c^	16.4 ± 0.69 ^c^
IL-6	99.00 ± 5.06	97.7 ± 6.06	164.7 ± 6.92 ^ab^	114.5 ± 8.24 ^abc^	110.0 ± 7.24 ^c^	105.2 ± 6.43 ^c^
TNF-α	2365.5 ± 53.63	2351.8 ± 45.96	3268.5 ± 46.47 ^ab^	2733.5 ± 30.09 ^abc^	2526.0 ± 31.55 ^c^	2410.3 ± 41.37 ^c^

Data are mean ± standard deviation. OLE: olive leaves extract, Cis: cisplatin, Cis + OLE: cisplatin + olive leaves extract. Cis + CM-MSC: cisplatin+ conditioned media mesenchymal stem cells; Cis + MSC: cisplatin + mesenchymal stem cells. IL-1β: interleukin-1β (ng/g tissue); IL-6: interleukin-6 (pg/g tissue); TNF-α: tumor necrosis factor-α (pg/g tissue). ^a^: significant compared to the control group; ^b^: significant compared to OLE group; ^c^: significant compared to Cis group. *n* = 6/group.

**Table 6:** Effects of olive leaf extract (OLE), mesenchymal stem cells (MSC), CM-MSC, cisplatin and their combination on 8-OH-dG, LDH, lysozyme activity and NO of rats.

Parameters	Control	OLE	Cis	Cis + OLE	Cis + CM-MSC	Cis + MSC
8-OH-dG (ng/mL)	4.15 ± 0.10	4.47 ± 0.28	17.18 ± 0.75 ^ab^	9.23 ± 0.67 ^c^	7.93 ± 0.48 ^c^	6.02 ± 0.69 ^c^
LDH (µ/L)	2280 ± 51	2323 ± 64	3912 ± 55 ^ab^	2620 ± 63 ^c^	2433 ± 85 ^c^	2403 ± 78 ^c^
Lysozyme activity (µg/mL)	284.33 ± 4.76	289.50 ± 5.32	226.83 ± 4.58 ^ab^	265.17 ± 4.17 ^c^	269.83 ± 4.62 ^c^	279.50 ± 6.22 ^c^
NO (ng/mL)	8.55 ± 0.55	8.95 ± 0.29	19.50 ± 2.43 ^ab^	13.62 ± 1.23 ^c^	11.70 ± 1.10 ^c^	9.63 ± 1.20 ^c^

Data are mean ± standard deviation. OLE: olive leaves extract, Cis: cisplatin, Cis + OLE: cisplatin+ olive leaves extract. Cis + CM-MSC: cisplatin + conditioned media mesenchymal stem cells; Cis + MSC: cisplatin + mesenchymal stem cells. 8OH-dG: 8-hydroxy-2-deoxyguanosine; LDH: lactate dehydrogenase; NO: nitric oxide. ^a^: significant compared to the control group; ^b^: significant compared to OLE group; ^c^: significant compared to Cis group. *n* = 6/group.

**Table 7:** The mean and standard deviation (SD) of the optical density of anticytokeratin immunoreaction activity in the reticuloepithelial cells of the thymus in the different experimental groups.

Parameters	Control	OLE	Cis	Cis + OLE	Cis + CM-MSC	Cis + MSC
Mean ± SD	166.1 ± 24.2	170.3 ± 40.4	188.4 ± 40.8 *	173.9 ± 29 *#	175.2 ± 34 *#	174.4 ± 32.1 *#

Statistically significant compared to control group (*) and cisplatin group (#); *p* < 0.05. OLE: olive leaves extract, Cis: cisplatin, Cis + OLE: cisplatin + olive leaves extract. Cis + CM-MSC: cisplatin + conditioned media mesenchymal stem cells; Cis + MSC: cisplatin + mesenchymal stem cells.

**Table 8:** The mean and standard deviation (SD) of the optical density of PCNA immunoreaction activity in the spleen in the different experimental groups.

Parameters	Control	OLE	Cis	Cis + OLE	Cis + CM-MSC	Cis + MSC
Mean ± SD	175.8 ± 26.3	173.4 ± 27.7	149.3 ± 43.4 *	164.5 ± 30.2 *#	164.7 ± 37.9 *#	165.2 ± 32.1 *#

Statistically significant compared to control group (*) and cisplatin group (#); *p* < 0.05. OLE: olive leaves extract, Cis: cisplatin, Cis + OLE: cisplatin + olive leaves extract. Cis + CM-MSCs: cisplatin + conditioned media mesenchymal stem cells; Cis + MSC: cisplatin + mesenchymal stem cells.

## Data Availability

The raw data supporting the conclusion of this article will be made available by the authors, on reasonable request.
